# Integrating Metabolomics and Transcriptomics to Analyse and Reveal the Regulatory Mechanisms of Mung Bean Polyphenols on Intestinal Cell Damage Under Different Heat Stress Temperatures

**DOI:** 10.3390/nu17010088

**Published:** 2024-12-29

**Authors:** Yuchao Feng, Shu Zhang, Decheng Suo, Tianxin Fu, Ying Li, Zetong Li, Changyuan Wang, Xia Fan

**Affiliations:** 1 Institute of Quality Standards and Testing Technology for Agro-Products of Chinese Academy of Agricultural Sciences, Beijing 100081, China; fengyuchao0321@126.com (Y.F.); suodecheng@caas.cn (D.S.); lilizetong@163.com (Z.L.); 2 College of Food, Heilongjiang Bayi Agricultural University, Daqing 163319, China; zshu996@163.com (S.Z.); futianxin940615@163.com (T.F.); byndliying@163.com (Y.L.)

**Keywords:** mung bean polyphenols, heat stress, gut, multi-omics, mechanism

## Abstract

Background/Objectives: Polyphenols represent a new strategy of dietary intervention for heat stress regulation. Methods: The metabolic and genetic effects of three heat stress-regulated mung bean polyphenols on mouse small intestinal epithelial Mode-k cells were investigated by metabolomics–transcriptomics correlation analysis at different heat stress levels. Results: Lipid metabolism, energy metabolism, and nervous system pathways were the key metabolic regulatory pathways. Under the heat stresses of 39 °C, 41 °C, and 43 °C, the key pathways regulated by mung bean polyphenols on intestinal epithelial Mode-k cells were choline metabolism, pyrimidine metabolism, and the retrograde endorphin signalling pathway in cancer, respectively. FoxO, Rap1, and PI3K-Akt signalling pathways were the key environmental regulatory signalling pathways. Mung bean polyphenols can alleviate heat stress-induced cells at 39 °C by inhibiting cell apoptosis and promoting lipid and amino acid accumulation. Mung bean polyphenols can alleviate the threat of cell death caused by heat stress at 41 °C by regulating heat shock proteins, inhibiting mitochondrial function and some nerve disease-related genes. The threat of cell death by heat stress at 43 °C can be alleviated by regulating nerve-related genes. Conclusions: This study confirmed that mung bean polyphenols can regulate heat stress. The results provide a reference for analysing the mechanism of dietary polyphenol regulating heat stress.

## 1. Introduction

Heat is a specific and important environmental stressor. Heat stress is the sum of non-specific responses in humans or animals to stimulation by excessively high temperatures that exceed their thermoregulatory capacity, which can induce oxidative stress and immune imbalance in the body [1], and, in severe cases, can even lead to death. Heat stress has become a major type of stress with serious social and economic impacts. High-temperature stress has continued to increase in recent years, and reducing the impact of heat stress on humans and animals has become a hot topic in current scientific research. An increasing number of studies have shown that heat stress initially reduces dietary intake and nutrient absorption and then affects the metabolic level of the body, leading to disorders in body functions [2,3]. The intestine is an important nutrient absorption site, as well as the largest organ of immunity in the body, and is sensitive to heat [4]. Heat stress can lead to the disruption of the integrity of the intestinal barrier, which is dependent on intestinal epithelial cells for its integrity and function, and cause an imbalance in intestinal flora [5,6]. Some nutrients have a regulatory effect on intestinal barrier integrity and beneficially regulate the structure, function, and immune response of the intestinal barrier through the intestinal contents [7,8]. Therefore, the alleviation of heat stress through dietary interventions has attracted the attention of researchers. In addition to amino acids, vitamins, minerals, etc., intervention by anti-stress components provides a valuable strategy for intestinal heat stress protection.

In recent years, dietary polyphenols have received renewed attention for their anti-stress effects in animal models and human trials [9]. Furthermore, they show potential in treating chronic intestinal diseases and intestinal flora regulation, and some of the mechanisms have been reported. Polyphenols represent a new strategy for dietary intervention in heat stress modulation [10,11,12]. Few studies on dietary interventions for heat stress prevention and control related to humans have been conducted. As animals are more sensitive to temperature than humans, more studies have been conducted on livestock and poultry than on humans to adopt dietary intervention for heat stress prevention and control [13]. Polyphenols modulate different cellular functions such as neutralising the effects of oxidative stress, inflammation, and apoptosis. Resveratrol, curcumin, and epigallocatechin gallate are the more researched and effective polyphenols in modulating heat stress [14,15,16]. Studies have also found that the mechanism of action varies depending on the polyphenol type. For example, polyphenols act as free radical scavengers according to their chemical structure to eliminate heat stress-induced oxidative damage or improve the antioxidant enzyme system by up-regulating antioxidant response pathways such as transcription factor-mediated antioxidant enzymes or by blocking the action of some enzymes that directly produce O_2_^-^ (e.g., xanthine oxidase and protein kinase C). The mechanisms of action also include mitigating the effects of heat stress by improving the activity of antioxidant enzymes and non-enzymatic systems or modulating heat shock protein expression [17,18,19,20]. However, the majority of previous studies have focused on the effects of improvements in physicochemical indexes and apparent behaviours under heat stress, and the mechanism of how to play a regulatory role in the organism remains unclear.

Mung bean (*Vigna radiata* L.) is a traditional Chinese food used to relieve heatstroke and is the preferred ingredient for heat stress dietary intervention. The statement that “its active components against heatstroke may be mainly dominated by polyphenolic antioxidants” is well accepted [21]. Although mung bean polyphenols are rich in variety and content, the key active polyphenols that play a role in the regulation of heat stress and their regulatory molecular mechanisms have not yet been revealed. The research group previously established a method for the detection of 20 mung bean polyphenols based on ultra-high performance liquid chromatography–high-resolution orbit trap liquid mass spectrometry (UHPLC-QE-HF-HRMS) using a combination of non-targeted and targeted methods [22]. The polyphenols were determined in terms of their content, Diphenyl picryl hydrazinyl radical and 2,2′-azino-bis(3-ethylbenzothiazoline-6-sulfonic acid) radical scavenging ability, ability to regulate heat shock protein 70 (HSP70) levels in the mouse small intestinal epithelial Mode-k cell heat stress model, and the effect of cellular epimorphic damage regulation. Vitexin, orientin, and caffeic acid are the three main polyphenolic fractions with heat stress modulatory effects in mung bean [22]. These three compounds are also present in other plants; however, only mung bean has a heat stress modulating effect. Therefore, it is speculated that vitexin, orientin, and caffeic acid are a synergistic group of active components in mung bean that have a heat stress modulating effect. It has also been shown that orientin and vitexin coexist in *Polygonum orientale* L. and *Trollius chinensis Bunge* (i.e., two medicinal plants with heat-clearing efficacy), which are synergistic components [23]. Although caffeic acid has not been reported in studies on heat stress regulation, it is one of the six key components in the Reyanning Keli, and also exhibits inflammatory and antioxidant regulation [24,25]. Therefore, this study was based on the mouse small intestine epithelial Mode-k cell heat stress model and involved metabolomics and transcriptomics correlation analysis to comprehensively explore the three polyphenol mixture components of intestinal cells under different degrees of heat stress (39 °C, 41 °C, and 43 °C) of the regulatory mechanism. The results preliminarily reveal the underlying reasons why mung bean polyphenols relieve heatstroke and provide theoretical references for analysing the mechanism of polyphenols in regulating heat stress.

## 2. Materials and Methods

Cells, instruments, and materials. Mouse intestinal epithelial Mode-k cells were purchased from Bei Na Biotechnology Co. (Beijing, China). The following instruments were used: micropipettes (Sartorius, Göttingen, Germany); clean bench (SW-CJ-1FD; Layte, Nantong, China); CO_2_ cell incubator (Thermo Fisher Scientific, WML, Waltham, MA, USA); MF52-N inverted microscope (Guangzhou Mshot Optoelectronic Technology Co., Guangzhou, China); L3-5K low-speed centrifuge (Ke-Cheng Technology Co., Taiwan, China); HH-2water bath (Changzhou Aohua Instrument Co., Guangzhou, China); BioTek ELx800 enzyme marker (Turner BioSystems, Sunnyvale, CA, USA); CytoFLEX flow cytometer (Beckman, Brea, CA, USA); UHPLC-Q Exactive HF-X ultraperformance liquid chromatography–tandem Fourier-transformed mass spectrometer (Thermo Fisher Scientific, Guangzhou, China); NewClassic MF MS105DU electronic balance (Mettler, Zurich, Switzerland); GeneAmp^®^ 9700 polymerase chain reaction (PCR) cycler (ABI, Austin, Benton Harbor, MI, USA); NovaSeq 6000 sequencer (Illumina, San Diego, CA, USA); and DYY-6C electrophoresis power supply (Beijing Liuyi Instrument Factory, Beijing, China).

The following materials were used: penicillin–streptomycin solution (100×); 0.25% trypsin solution (containing ethylenediaminetetraacetic acid dissolved in phosphate buffered saline [PBS]); Dulbecco’s modified Eagle’s medium (Wuhan Procell Life Sciences Co., Wuhan, China); anti-β-actin antibody (Beijing Boao Sen Biotechnology Co., Beijing, China); anti-HSP70 antibody (3A3) (Abcam, Cambridge, UK); horseradish peroxidase-labelled IgG (Biosharp, Chongqing, China); foetal bovine serum (ExCell Bio, Suzhou, China); thiazolyl blue (MedChemExpress, Shanghai, China); dual antibody (Thermo Fisher Scientific, Guangzhou, China); vitexin, orientin, and caffeic acid (Shanghai Yuanye Biotechnology Co., Ltd., Shanghai, China); library building kits (NEXTFLEX Rapid DNA-Seq; Bio Scientific, Shanghai, China); sequencing kits (NovaSeq Reagent; Illumina, San Diego, CA, USA); pipettes (N13462C; Eppendorf, Hamburg, Germany); and cell culture dishes, 96-well cell culture plates, and cell culture flasks (Corning, NY, USA).

Cell culture and passaging. Mode-k cells were cultured in Dulbecco’s modified Eagle’s medium supplemented with 10% foetal bovine serum and 1% penicillin–streptomycin solution. For cell resuscitation, the Mode-k cells were removed from liquid nitrogen, immediately placed in a water bath at 37 °C, and gently shaken to dissolve the cryopreservative. The cells were then transferred to a centrifuge tube containing 5 mL of a medium and centrifuged at 1000 rpm at room temperature for 5 min. The supernatant was discarded, and the cells were collected and suspended in a complete medium containing 10% foetal bovine serum, spread onto Petri dishes, gently mixed, and incubated at 37 °C in the presence of 5% CO_2_ with saturated humidity.

The cells were passaged when they reached 80% density. The medium was discarded, the cells were washed with PBS, and 0.25% trypsin (1–2 mL) was then added to digest the cells for 1–2 min. The cells were observed under a microscope and digestion was considered complete when the cells were separated from each other and rounded. The trypsin was discarded, and a complete medium was added. The cells were gently shaken to achieve a single-cell suspension, passaged at a 1:3 ratio, and cultured at 37 °C in the presence of 5% CO_2_ with saturated humidity.

Modulation of the Mode-k cell heat stress model with mung bean polyphenols. The following groups were established: (1) 37 °C blank control group (CON): Mode-k cells were cultured normally without any treatment, and the culture time was the same as that of the heat stress group. (2) Heat stress model group (39 °C HS, 41 °C HS, and 43 °C HS): Mode-k cell splice plates were grown to approximately 80% of the density, and a medium was added to continue the culture for 12 h. Mode-k cells were then subjected to heat stress culture at 39 °C, 41 °C, and 43 °C for 6 h, followed by a 6 h recovery period at 37 °C. (3) Mung bean polyphenol heat stress modulation experimental group (39 °C DF, 41 °C DF, and 43 °C DF): Mode-k cells were grown to a density of approximately 80%, and a medium containing a mixture of three monomer mung bean polyphenols (vitexin, orientin, and caffeic acid at a mass ratio of 10:3:6 with a final concentration of 50 µL/mL) was added for a total of 12 h. The Mode-k cells were then placed in a heat stress incubator for 6 h at 39 °C, 41 °C, or 43 °C and then allowed to recover at 37 °C for 6 h. Then, the cells were collected as required and incubated at 39 °C, 41 °C, or 43 °C for 6 h and then allowed to recover at 37 °C for 6 h. Finally, the cells were collected as required for metabolomics and transcriptomics assays.

HSP70 electrophoresis. Total protein was extracted from 10^7^ cells. Proteins were separated by electrophoresis on denaturing polyacrylamide gels and blotted onto polyvinylidene difluoride membranes. The membranes were incubated with Tris-buffered saline (TBS) containing 5% skimmed milk powder at room temperature for 2 h and then probed with diluted primary antibodies (mouse anti-human β-actin and HSP70 antibodies) at 4 °C overnight. The following day, after three washes with 0.5% Tween-20/TBS at room temperature, the membranes were incubated with the secondary antibody (horseradish peroxidase-labelled goat anti-mouse IgG antibody) for 2 h. The membranes were then washed again three times with 0.5% Tween-20 in TBS at room temperature, and specific bands were detected using an ECL chemiluminescence kit (Shanghai Bangjing Industrial Co., Ltd., Shanghai, China).

Metabolomic analysis. Metabolomic analysis was performed * a UHPLC-Q Exactive HF-X ultraperformance liquid chromatography–tandem Fourier-transformed mass spectrometer. Raw mass spectrometry data were filtered for missing values, which were simulated. The data were then normalised, quality-controlled, and converted. The data were compared with the Kyoto Encyclopedia of Genes and Genomes (KEGG) and Human Metabolome Database (HMDB) databases to obtain metabolite annotation information. The data were then subjected to multivariate statistical analyses, including principal component analysis (PCA) and orthogonal partial least squares–discriminant analysis (OPLS-DA) using the ROPLS package in R (Version1.6.2). To screen for differential metabolites, we used univariate statistical analysis (*t*-tests) combined with multivariate statistical analysis (OPLS-DA/PLS-DA). Differential metabolites were screened out based on *p* < 0.05 and variable importance on projection VIP > 1. SciPy in Python was used for differential metabolite metabolic pathway enrichment analysis and VIP value analysis. Based on metabolite comparison to KEGG compound IDs, metabolic pathway information was obtained, and hierarchical clustering analysis was performed. Based on the metabolite expression information in different samples, the distance of metabolites or samples was calculated, and the metabolites or samples were then classified using an iterative approach. VIP value analysis of the enriched metabolic pathways was performed using ropls in R.

Cellular transcriptomics analysis. Total RNA was extracted from the cells (QIAzolLysis Reagent; Qiagen, NY, USA), and the RNA concentration and purity were determined using a Nanodrop2000 (Thermo Fisher Scientific, Waltham, MA, USA). RNA integrity was assessed by agarose gel electrophoresis, and RNA integrity number (RIN) values were determined using an Agilent 2100 instrument (Agilent Technologies, Santa Clara, CA, USA). Single builds required total RNA ≥ 1 µg, a concentration ≥ 35 ng/µL, OD260/280 ≥ 1.8, and OD260/230 ≥ 1.0. mRNA was enriched using oligo (dT) magnetic beads and digested in fragmentation buffer to obtain approximately 300 bp fragments, which were isolated by magnetic bead screening. The fragments were subjected to reverse cDNA synthesis using reverse transcriptase and six-base random primers (random hexamers). cDNA sticky ends were filled using an End Repair Mix, followed by the addition of an A base at the 3′ end. Libraries were enriched by PCR amplification in 15 cycles. PCR products were subjected to 2% agarose gel to recover the target bands. We used TBS380 (PicoGreen) for quantification. Clusters were generated by bridge PCR amplification on cBot, followed by sequencing on a NovaSeq 6000 platform (read length 2 × 150 bp). The reads were matched with the GRCm39 reference genome (http://asia.ensembl.org/Mus_musculus/Info/Index, accessed on 25 December 2024) for each sample separately. Gene expression and differential expression analyses were performed using kallisto (version 0.46.2), Salmon (version 1.3.0), RSEM (version 1.3.3), DESeq2 (version 1.24.0), edgeR (version 3.24.3), DEGSeq (version 1.38.0), Limma (version 3.38.3), and NOISeq (version 2.18.0). Differential expression was defined based on |log_2_(fold change)| ≥ 1 and *p* < 0.05. Genes were functionally annotated using nine databases including gene ontology (GO), KEGG, nr, EggNOG, Swiss-Prot, Pfam, Reactome, DO, and DisGeNet, using Blast2go (version 2.5), HMMER (version 3.2.1), KOBAS (version 2.1.1), and goatools (version 0.6.5). The data were analysed on the online Majorbio Cloud Platform (www.majorbio.com, accessed on 25 December 2024).

## 3. Results

### 3.1. Expression of Mung Bean Polyphenol HSP70 mRNA and Protein at Different Heat Stress Temperatures

**Figure 1:**
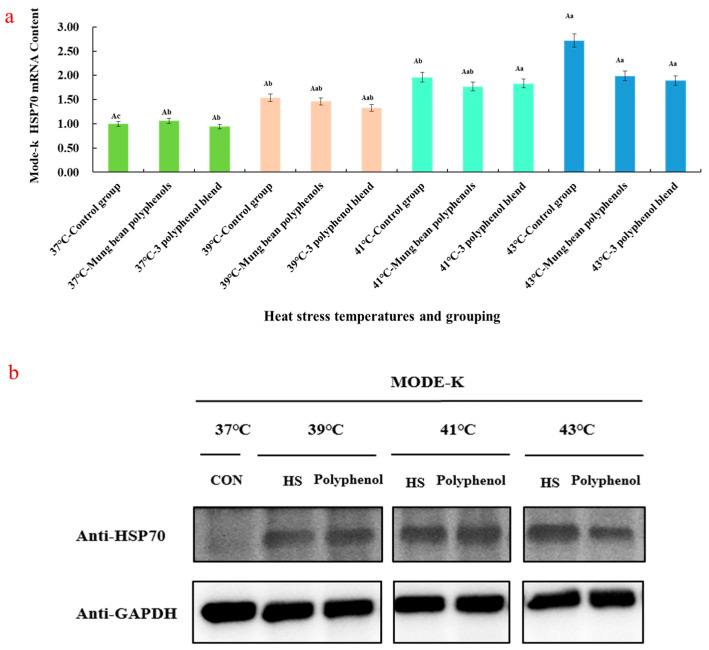
Effect of heat stress and mung bean polyphenol modulation on HSP70 mRNA and protein expression in Mode-k cells. (**a**) Regulation of HSP70 mRNA levels in Mode-k cells by mung bean polyphenols under different heat stress degrees (In the significance analysis of the bar chart, “A” represents the significant difference within the group; ’a, b, c’ represents inter group differences.); (**b**) effect of mung bean polyphenols on the expression level of HSP70 protein in Mode-k cells under different heat stress temperatures.

Heat stress induces heat shock protein accumulation in cells [26]. Figure 1a shows the regulation of HSP70 mRNA levels in Mode-k cells by mung bean mixed polyphenols (free and bound phenols) as well as the three polyphenol mixtures of vitexin, orientin, and caffeic acid (herein referred to as mung bean polyphenols) under different heat stress degrees. Compared with the 37 °C control group, the HSP70 mRNA level increased with temperature. Moreover, both mung bean mixed polyphenols and mung bean polyphenols reduced the level of HSP70 mRNA under heat stress, and mung bean polyphenols were more effective in reducing the level of HSP70 mRNA. Figure 1b shows the effect of mung bean polyphenols on the expression level of HSP70 protein in Mode-k cells under different heat stress temperatures. As seen from the colour depth of the bands in the electrophoresis graphs, the three temperatures of 39 °C, 41 °C, and 43 °C obviously increased HSP70 protein expression. Moreover, the higher the temperature, the darker the colour of the electrophoretic bands, indicating that the increase in protein expression levels between different comparison groups was more intuitive. The mung bean polyphenol-regulated group at different temperatures became lighter in colour compared with the HS group, indicating that mung bean polyphenols reduced the heat stress-induced expression of HSP70 protein levels; i.e., mung bean polyphenols had a regulatory effect on heat stress, which was more obvious at higher heat stress temperatures.

### 3.2. Metabolomic Impact Analysis of Mode-k Cells Modulated by Heat Stress at Different Temperatures by Mung Bean Polyphenols

#### PLS-DA Analysis and Differential Metabolite Screening

**Figure 2:**
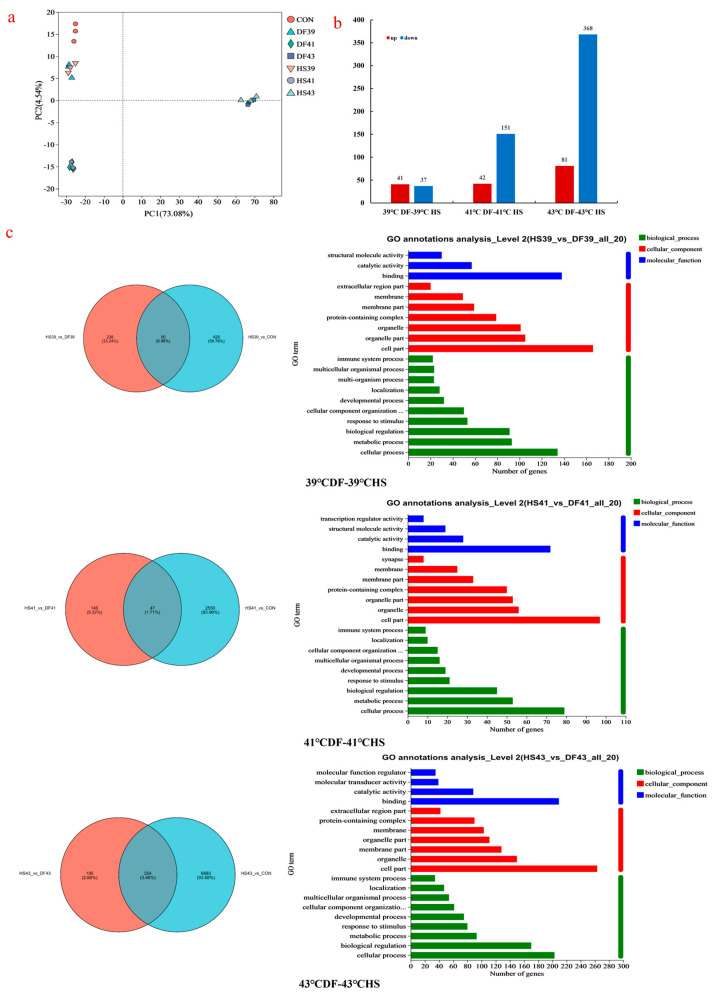
PLS-DA scatterplot, hierarchical clustering analysis of differential metabolites, and analysis of VIP values. (**a**) PLS-DA scatter plot for different treatment groups. (**b**) Column chart of changes in the quantity of differential metabolites (content up/down regulation) in different treatment groups. (**c**) Hierarchical clustering analysis results of differential metabolites and Venn Diagram plots. Note, in the PLS-DA scores are plotted with “Component 1” first principal component explanations. The horizontal coordinate in the volcano plot is the value of the fold change in the difference in metabolite expression between the two groups, i.e., “log2FC”. The vertical coordinate is the value of the statistical test for the difference in metabolite expression change, i.e.—the “log10(*p*-value)” value. Each point in the graph represents a specific metabolite, and the size of the point indicates the VIP value. Red points indicate significantly upregulated metabolites, blue points indicate significantly downregulated metabolites, and grey points are non-significantly different metabolites. Each column in the hierarchical cluster analysis heatmap represents one sample, and each row represents one metabolite. The colours in the graph indicate the relative expression size of the metabolites in the group of samples; please see the numerical labels under the colour bars at the bottom right for the specific expression size trends. The left side is the dendrogram of metabolite clustering, and the right side is the name of the metabolite. The left side of the VIP value graph is the metabolite VIP bubble graph. The y-axis indicates the metabolite, and the x-axis is the VIP value. Metabolites are arranged according to the size of the VIP value, from top to bottom. On the right side is the metabolite expression heatmap. Each column represents a sample, with the sample name below. Each row represents one metabolite. The colour indicates the relative expression size of the metabolite in that group of samples, and the numerical magnitude is shown in the gradient colour block.

The PLS-DA scatter score plots of Mode-k cells from the control group, heat stress groups at different temperatures, and mung bean polyphenol-regulated groups at different temperatures are shown in Figure 2a. The three heat stress temperatures in the confidence interval had their respective sample aggregation areas in the mung bean polyphenol group. This indicated that the effects of heat stress at different temperatures on the metabolism of Mode-k cells varied significantly. At the same heat stress temperature, the mung bean polyphenol group was also more strongly distinguished from the heat stress group, suggesting that mung bean polyphenols also affected heat stress cell metabolism. The detected metabolite types included eight types of carbohydrates, hormones and transmitters, lipids, nucleic acids, organic acids, peptides, steroids, vitamins, and cofactors. In terms of quantity, the cellular metabolites contained more lipids (mainly phospholipid and fatty acid substances) and peptides (mainly amino acid substances and cofactors). The mung bean polyphenol group was screened for differential metabolites in comparison with the heat stress group (the screening conditions were VIP > 1, *p* ≤ 0.05). The results showed that 105 (Table S1), 111 (Table S2), and 71 (Table S3) differential metabolites were screened in the 39 °C DF-39 °C HS, 41 °C DF-41 °C HS, and 43 °C DF-43 °C HS groups, respectively. The quantitative changes in the regulation of differential metabolite content are shown in Figure 2b. The differential metabolites were mainly lipids, such as phospholipids and alkanes, and several organic acids, steroids, vitamins, and cofactors. Mung bean polyphenols had fewer differential metabolites upregulated in content after heat stress modulation at 39 °C, and more differential metabolites were upregulated in content after heat stress modulation at 41 °C and 43 °C.

The hierarchical clustering analysis results of differential metabolites and Venn Diagram are shown in Figure 2c. The metabolic regulation of heat stress in Mode-k cells by mung bean polyphenols showed inverse regulation of the differential metabolite content, as shown by the colour changes in the heat stress and mung bean polyphenol groups at different temperatures. The colour comparison between the CON and mung bean polyphenol groups (Figure 2c) showed that the addition of mung bean polyphenol enabled the return of the levels of some metabolites with content changes induced by heat stress to normal levels. This indicated that mung bean polyphenols had the ability to regulate heat stress metabolism. Furthermore, the levels of some differential metabolites were different from those of the CON and HS groups, indicating that mung bean polyphenols also caused changes in other metabolic processes and played a role in heat stress regulation. Comparative analysis of differential metabolites in the three groups screened out four shared key metabolic markers: DG (10:0/0:0/20:5[6E,8Z,11Z,14Z,17Z]-OH[5]), PC(14:0/14:0), chelidonine, and apigenin.

**Figure 3:**
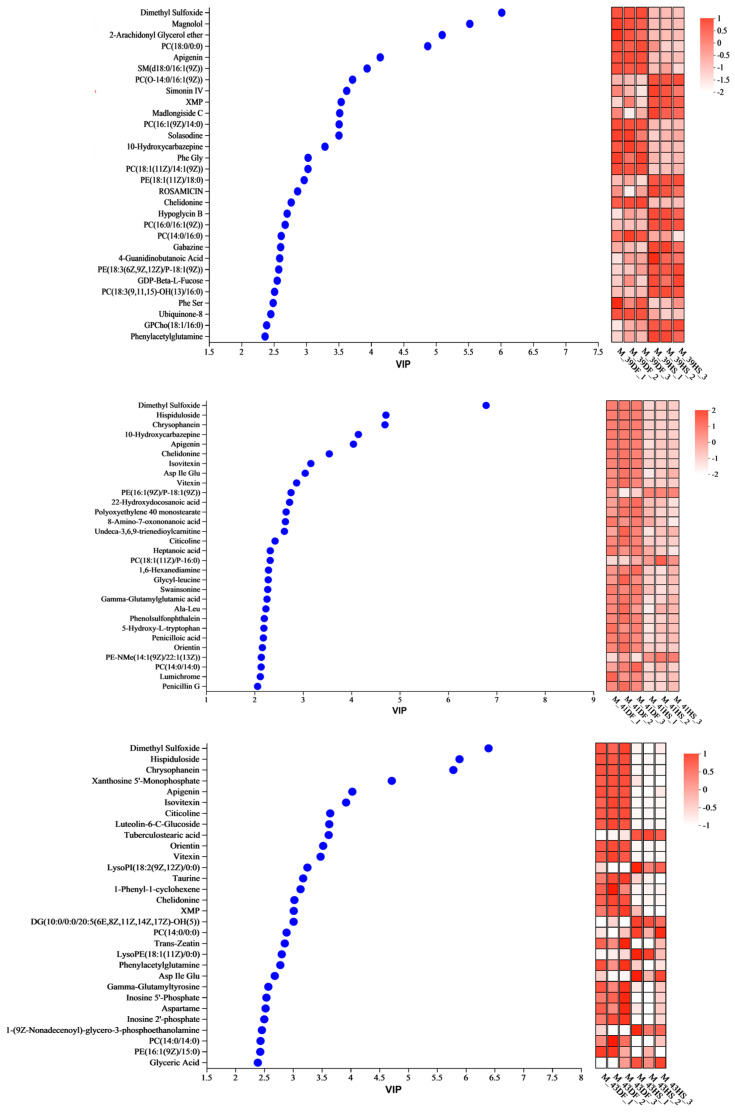
Metabolic analysis of VIP values in different comparison groups. Note, in the above figure, the left side is the dendrogram of metabolite clustering, and the right side is the name of the metabolite. The left side of the VIP value graph is the metabolite VIP bubble graph. The y-axis indicates the metabolite, and the x-axis is the VIP value. Metabolites are arranged according to the size of the VIP value, from top to bottom. On the right side is the metabolite expression heatmap. Each column represents a sample, with the sample name below. Each row represents one metabolite. The colour indicates the relative expression size of the metabolite in that group of samples, and the numerical magnitude is shown in the gradient colour block.

VIP values were analysed for the top 30 differential metabolites (Figure 3). From the types of differential metabolites, mung bean polyphenols that regulated heat stress at 39 °C were mainly phospholipids and amides. The VIP value showed that the regulation degree of mung bean polyphenols on cell metabolism under heat stress was much stronger than that of heat stress on cell metabolism. Comparative analysis revealed that PC (18:0/0:0) and phenylalanine glycine were the downregulated substances in content after heat stress. Mung bean polyphenols were the upregulated substances in content after modulation. Phenylacetylglutamine, hypoglycine B, PC (O-14:0/16:1 [9Z]), gabazine, and 4-guanidinobutyric acid were upregulated in content after heat stress and downregulated in content after mung bean polyphenol modulation (i.e., they were the metabolic markers of mung bean polyphenol heat stress modulation at 39 °C) and fatty acids and amino acids were the main metabolic markers at 41 °C. As shown by the magnitude of the VIP values, the effect of heat stress on cellular metabolism at 41 °C was slightly stronger than that of mung bean polyphenols on heat stress regulation. Mainly lipids, amides, and polyphenols were the main metabolic markers at 43 °C. Mung bean polyphenol modulation resulted in highly significant increases in 22 metabolites. The effect of heat stress on cellular metabolism was slightly stronger than that of mung bean polyphenols on heat stress modulation, as shown by the magnitude of the VIP values. Comparative analyses revealed that the taurine content was upregulated after heat stress and downregulated after mung bean polyphenol treatment. The contents of 1-(9Z nonadienoyl)-glycerol-3-phosphate ethanolamine and LysoPI (18:2 [9Z, 12Z]/0:0) were downregulated after both heat stress and mung bean polyphenol treatment. Inosine 2′-phosphate was upregulated after both heat stress and mung bean polyphenol treatment.

### 3.3. Enrichment Analysis of the KEGG Metabolic Pathways Regulated by Heat Stress in Mung Bean Polyphenols

**Figure 4:**
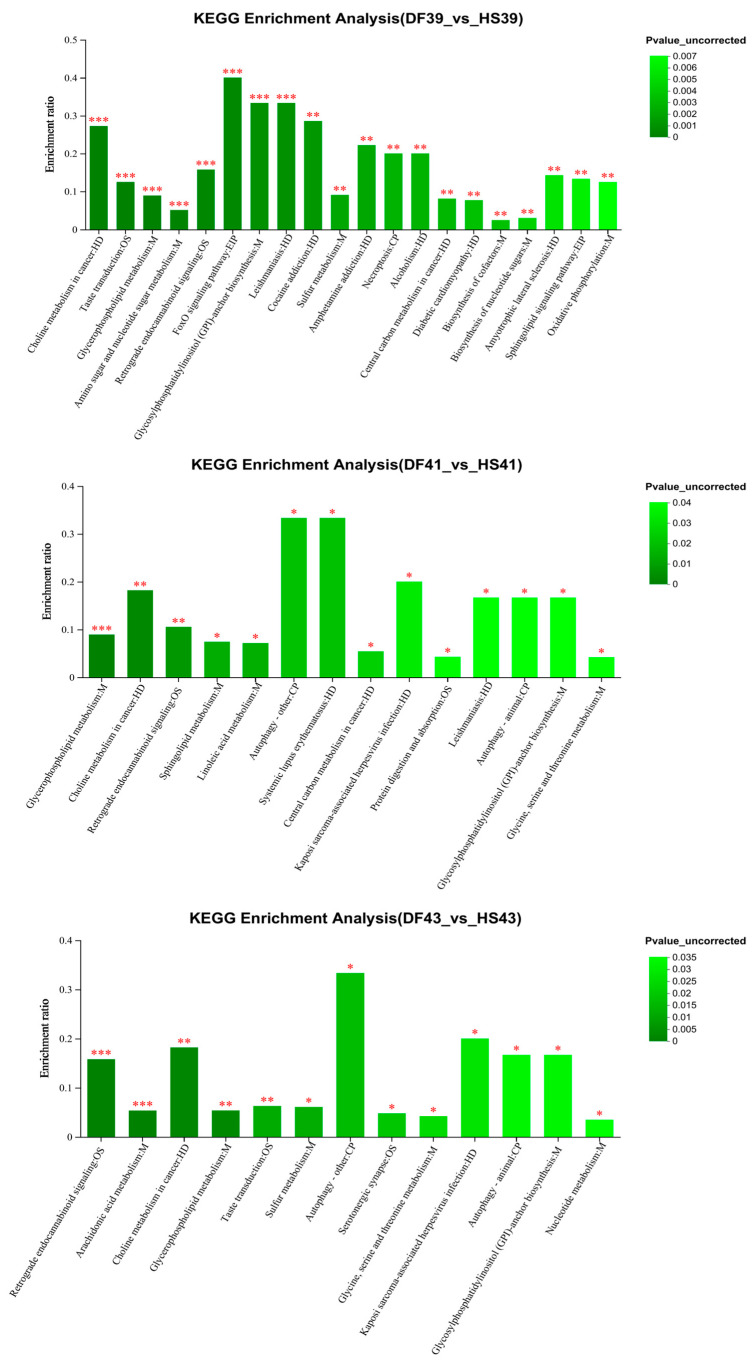
Results of enrichment analysis of KEGG metabolic pathways regulated by different heat stress temperatures for mung bean polyphenols. * Represents *p* < 0.05, ** represents *p* < 0.01, *** represents *p* < 0.001.

The screened differential metabolites were analysed for KEGG pathway enrichment (Figure 4). The heat stress group was analysed in comparison with the mung bean polyphenol group, and 20, 14, and 13 differential metabolic pathways were enriched at 39 °C, 41 °C, and 43 °C, respectively (Tables S4–S6). The key pathways of mung bean polyphenols at 39 °C for heat stress regulation included the FoxO signalling, central carbon metabolism, sugar biosynthesis and metabolism, and cardiovascular disease pathways. The metabolites involved in pathway regulation were mostly lipids and nucleic acids. The key pathways of mung bean polyphenols at 41 °C for heat stress regulation included the retrograde endogenous cannabinoid signalling pathway associated with the nervous system, the cysteine and methionine metabolism pathway, and the protein digestion and absorption pathway. The metabolites involved in pathway regulation were mostly lipids. The key pathways of mung bean polyphenols at 43 °C for heat stress regulation included central carbon metabolism in cancer, the sensory system-related taste transduction pathway, the neurotrophin signalling pathway, and sphingolipid metabolism. The metabolites involved in pathway regulation were mostly lipids and nucleic acids.

A total of four metabolic pathways shared by both heat stress and mung bean polyphenol regulation processes were screened (Table 1): choline metabolism, lipid metabolism, carbohydrate biosynthesis and metabolism, and neural system-related pathways. It can be inferred from the metabolites involved in the regulation of key pathways that the regulation of mung bean polyphenols on heat stress was closely related to lipid metabolism and accumulation.

### 3.4. Mode-k Cell Transcriptomic Analysis of Heat Stress Regulation of Mung Bean Polyphenols at Different Temperatures

#### 3.4.1. Gene Principal Component Analysis and Differential Gene Screening

**Figure 5:**
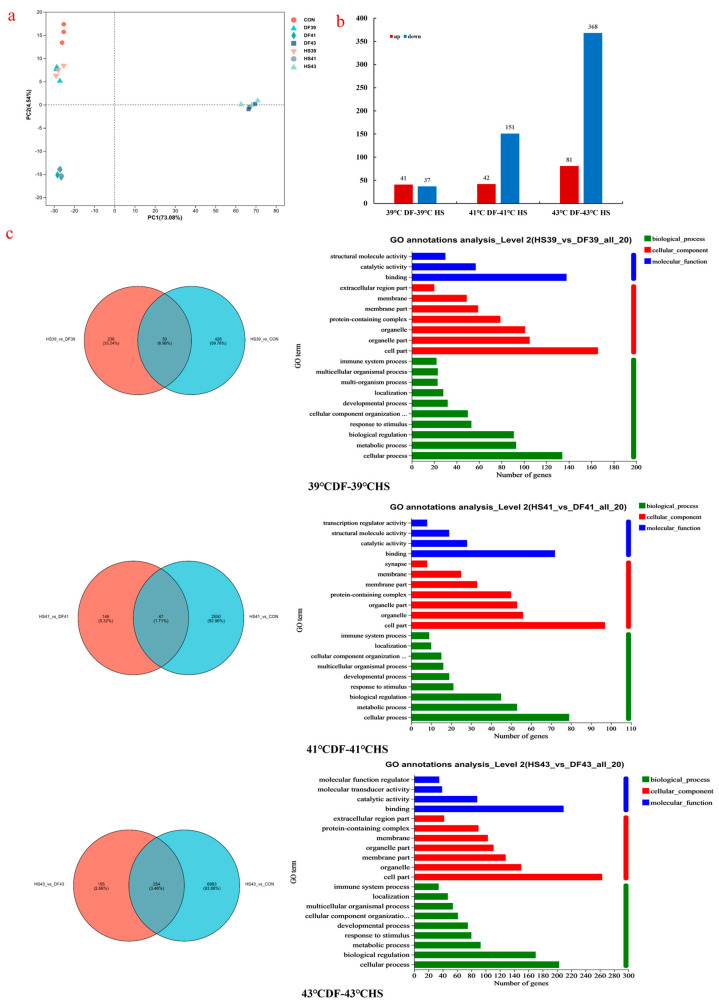
PCA score map of mung bean polyphenol heat stress regulation samples, statistical map of differential gene quantity, annotation of differential gene GO function, and analysis of gene regulatory pathway enrichment bubble chart.

The clean data of each sample reached ≥6.04 Gb. The clean reads of each sample were sequentially aligned with the designated reference genome, with alignment rates ranging from 93.55 to 95.5%. Figure 5a shows the PCA results for gene expression. The dispersion degree within gene sample point groups under different heat stress temperatures was very small, with each having its own aggregation zone. There was considerable distance between sample point groups under the different temperature heat stress and mung bean polyphenol groups, and the distance from the control group increased with temperature, indicating that heat stress caused significant differences in gene expression in Mode-k cells and that the higher the temperature, the greater the difference. Polyphenol treatment of mung beans can affect gene expression. Based on the quantitative expression results, inter-group differential gene analysis was performed to determine genes with differential expression between two sample groups. The statistical results are shown in Figure 5b, in which red and blue represent the number of upregulated and downregulated genes, respectively. Evidently, the gene expression changes under different temperature and heat stress conditions after treatment with mung bean polyphenols increased with temperature.

#### 3.4.2. Functional Annotation and Venn Analysis of Differentially Expressed Genes—GO and KEGG

Figure 5c shows Venn and functional annotation diagrams of three differentially expressed genes regulated by temperature heat stress and mung bean polyphenol heat stress. There were 50 (Table S7), 47 (Table S8), and 254 (Table S9) genes that underwent significant changes after heat stress at 39 °C, 41 °C, and 43 °C, respectively, and regulation of mung bean polyphenols. Except for a few genes, the differentially expressed genes between the three temperature heat stress and mung bean polyphenol groups were all reverse-expressed, indicating that mung bean polyphenols regulated heat stress by reverse-regulating differentially expressed gene expression. GO annotation was performed on differentially expressed genes and showed that the biological processes involved in heat stress and mung bean polyphenol regulation mainly included biological regulation; metabolic processes; cellular component organisation or biogenesis; cellular processes; developmental processes; and responses to stimuli, localisation, and other pathways. Molecular functions mainly involved binding, catalytic activity pathways, structural molecular activity, and molecular function regulator and transporter activity. Cellular components involved cellular components, organelles, organelle components, membranes, membrane components, and complex pathways containing proteins.

The genes with significantly reduced expression after heat stress regulation by mung bean polyphenols at 39 °C were screened and included Gm10233, Pdxp, Sall1, Lcat, Igkc, Mpc1-ps, Kansl2-ps, Gm5451, Gm27188, and Gm50092. Genes with significantly enhanced expression included H3c14, Tmem255a, Sorl1, Apol10a, Hspa1b, and 9230116L04Rik. Gene Gm42845 showed significantly decreased expression after heat stress regulation by mung bean polyphenols at 41 °C. Genes with significantly enhanced expression included Icam2, Crybb3, Bhlha15, Gm13192, Apc ps19230111E07Rik, Gm2244, Gm4540, Gm42457, Rpl10 ps5, and Gm50230. Genes with significantly reduced expression (with multiple changes ranging from 0.02 to 0.09) after heat stress regulation by mung bean polyphenols at 43 °C included Notum, Etv2, Prss41, Gm35546, Psme2b, Rapgef4os3 Rap, Gm50397, Gm45136, Gm7972, Gm49684, Slc25a54, Gm25547, Tmem53, Iqca1l, Olfr23, Rbbp8nl, Itcam, Cym, Gm52988, Gm45164, Gm7558, Myadml2, Alb, Klf15, Gm30505, Gm17189, Il13, Gm45837, Cdx4, Btla, Gm30593, Snord3b4, Olfr457, Gm49731, and Tcfl5. Genes with significantly enhanced gene expression were 1700006J14Rik and Gm52969.

#### 3.4.3. GO and KEGG Pathway Enrichment Analysis of Differentially Expressed Genes

**Figure 6:**
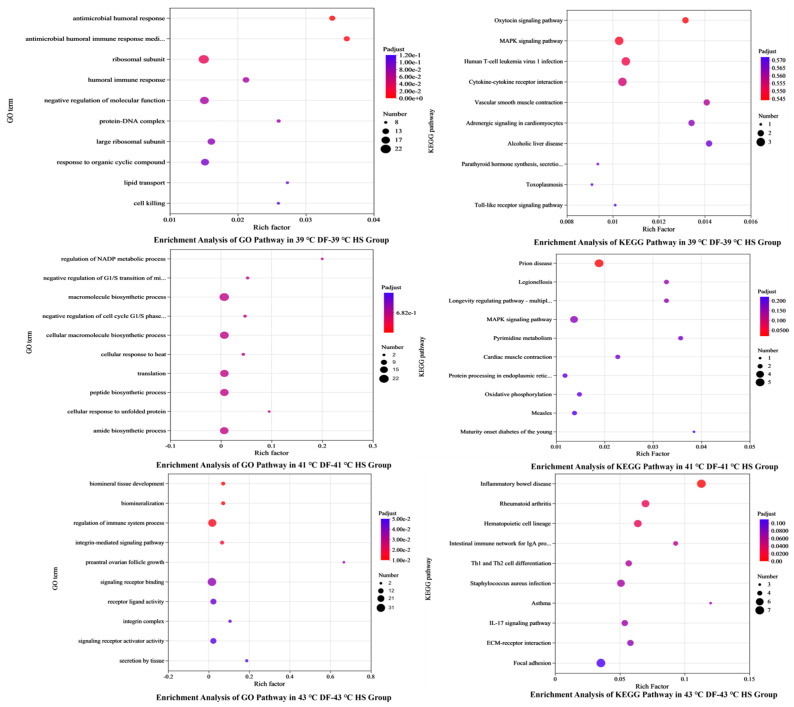
Analysis of gene regulatory pathway enrichment bubble chart.

GO and KEGG pathway enrichment analyses were performed on all differentially expressed genes to determine the regulatory functional pathways of differentially expressed genes and the main signal transduction pathways involved (i.e., the 10 pathways with the most significant *p*-value). The enrichment analysis results are shown in Figure 6. The differential genes regulated by mung bean polyphenols at 39 °C mainly involved pathways such as antimicrobial humoral response, antimicrobial peptide-mediated antimicrobial humoral immune response, ribosomal subunits, humoral immune response, negative regulation of molecular function, protein–DNA complexes, large ribosomal subunits, response to organic cyclic compounds, lipid transport, and cell killing. Those at 41 °C mainly involved pathways such as cell ribosomes, ribosome subunits, the regulation of NADP metabolism, transparent band receptor complexes, the negative regulation of G1/S phase transition in the mitotic cell cycle, the macromolecular biosynthesis process, the negative regulation of the G1/S phase transition in the cell cycle, the biosynthesis process of macromolecules, the cellular response to heat, translation, etc. Those at 43 °C mainly involved the development of biomineral tissues, biomineralisation, the regulatory processes of the immune system, integrin-mediated signalling pathways, pre-ovarian follicle growth, signal receptor binding, receptor ligand activity, integrin complexes, the activity of signal receptor activators, tissue secretion, etc. The key differential gene functions regulated by mung bean polyphenols varied at different temperatures.

Among the signal transduction pathways mainly involved in differential genes, the pathways involved in the regulation of differential genes by mung bean polyphenols at 39 °C were not significantly different overall. The 10 most significant pathways were determined (Table S10) and included the oxytocin signalling pathway, MAPK signalling pathway, Toll-like receptor signalling pathway, and cytokine–cytokine receptor interaction. The differential genes involved in the regulation by mung bean polyphenols at 41 °C involved MAPK signalling pathways, pyrimidine metabolism, and oestrogen signalling pathways (Table S11). At 43 °C, mung bean polyphenols regulated differentially expressed genes related to inflammatory bowel disease, the IgA-producing intestinal immune network, differentiation of Th1 and Th2 cells, IL-17 signalling pathways, and local adhesion of ECM receptor interactions (Table S12).

### 3.5. Metabolomic and Transcriptomic Correlation Analysis of Mode-k Cells Under Heat Stress Regulation by Mung Bean Polyphenols at Different Temperatures

**Figure 7:**
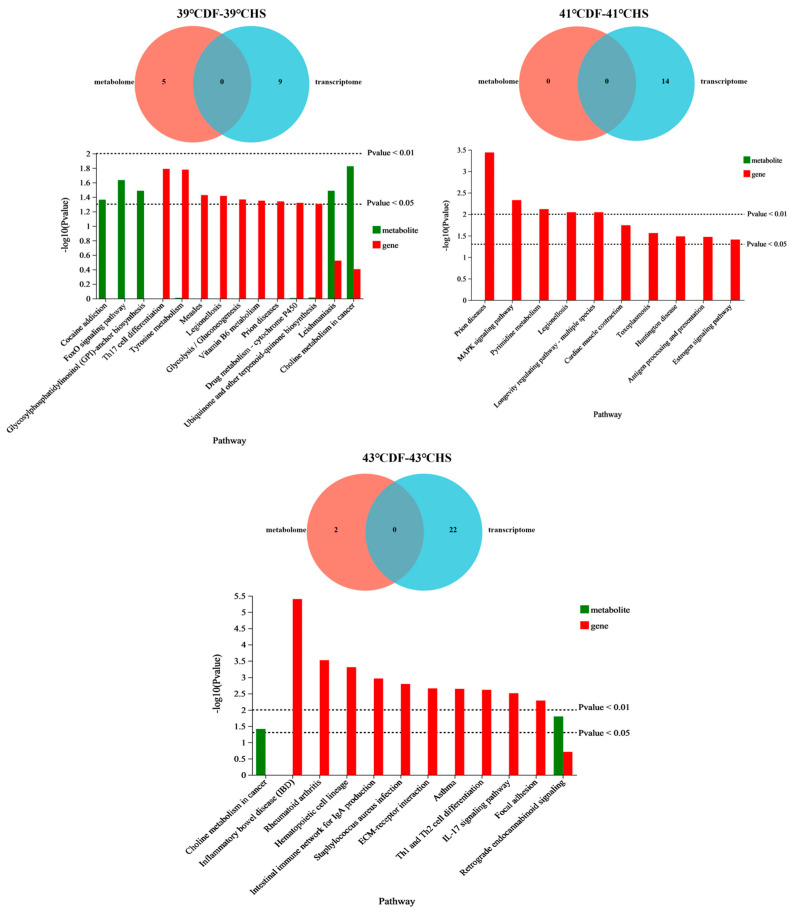
Annotated Venn and bar charts of differential genes and differential metabolite KEGG enrichment pathways regulated by mung bean polyphenols.

Under heat stress temperatures of 39 °C, 41 °C, and 43 °C, there were 14, 14, and 24 pathways with significant changes in gene and metabolic sets (*p* < 0.05) (Figure 7), respectively (Tables S13–S15 for detailed information).

Five metabolic pathways and nine gene pathways were significantly enriched after heat stress regulation by mung bean polyphenols at 39 °C. Among them, choline metabolism in cancer was the pathway with the most significant changes in metabolism and gene levels. There was one differentially expressed gene and 16 differentially expressed metabolites annotated in this pathway; the gene was Fos, which was downregulated in expression. The differentially expressed metabolites were all lipids and their derivatives, and their regulation is shown in Table 2. A further correlation analysis was conducted on one differentially expressed gene and 16 differentially expressed metabolites, and the Fos gene was only significantly correlated with GPCho (14:0/18:1) (*p* < 0.05). The Fos gene was significantly downregulated, and the content of the metabolite GPCho (14:0/18:1) in this pathway was significantly upregulated.

In addition, there were four pathways that were also pathways with simultaneous changes in metabolic and gene sets. The differential metabolites and differential gene changes were found in the leishmania pathway (phosphatidylserine [down], *N*-allyl neuraminate [downregulated], and Fos [downregulated]); tyrosine metabolism pathway (L-tyrosine [downregulated], Aldh3a1 [downregulated], and Adh1 [downregulated]); drug metabolism—the cytochrome P450 pathway (10-hydroxycarbazepine [upregulated], Aldh3a [downregulated], and Adh1 [downregulated]—and biosynthesis of urea and other terpenoid compounds quinones (L-tyrosine [downregulated] and Nqo1 [downregulated]).

After heat stress regulation at 41 °C, only 14 significantly altered gene pathways were enriched (Table 3). The main genes involved in the 14 pathways were Grin1, Hspa1b, Pde6a, Jun, Cox8a, EHspa1b, Ndufa4l2, Tymp, Hspa1l, and Entpd3. Entpd3 (26 times) and Hspa1l (21 times) were significantly upregulated and Pde6a (0.05 times) and Tymp (0.02 times) were significantly downregulated.

Retrograde endorphin signalling after heat stress modulation by mung bean polyphenols at 43 °C was a key pathway for simultaneous changes in the metabolic and gene sets. Three differential genes and three differential metabolites were annotated in the pathway: Gria4 (downregulated), mt-Nd3 (upregulated), and mt-Nd4l (downregulated). PE (16:1[9Z]/15:0), PC (14:0/14:0), and PGH2 contents were significantly upregulated (Table 4). The correlation analysis results are shown in Table 5. The mt-Nd3 gene was highly significantly negatively correlated with PC (14:0/14:0) and significantly negatively correlated with PGH2. The Gria4 gene was highly significantly positively correlated with PGH2 and significantly positively correlated with PE (16:1[9Z]/15:0).

Another pathway enriched in both the gene set and metabolism set is the neuroactive ligand–receptor interaction pathway, in which the metabolite is taurine (up), and the genes are Hrh4 (upregulated), Fpr2 (upregulated), Fpr1 (upregulated), Gpr83 (upregulated), Cckar (upregulated), Avp (upregulated), Grin3a (upregulated), and Gene Gria4 (downregulated). There were no significant correlations between genes and metabolites.

In summary, it was found that mung bean polyphenols had different effects on three key temperature regulation pathways. Heat stress regulation at 39 °C involved the FoxO signalling pathway in environmental information processing classification. Heat stress regulation at 41 °C involved the MAPK signalling pathway. Heat stress regulation at 43 °C involved the ECM receptor interaction, the calcium signalling pathway, the Rap1 signalling pathway, the cytokine–cytokine receptor interaction, the PI3K/Akt signalling pathway, and the neuroactive ligand–receptor interaction. Evidently, an increasing number of environment-related pathways were regulated as the degree of heat stress deepened. The metabolic pathways mainly involved carbohydrate biosynthesis and metabolism, amino acid metabolism, lipid metabolism, purine metabolism, pyrimidine metabolism, carbohydrate metabolism, cofactor and vitamin metabolism, and cytochrome P450 metabolism. The types of pathways involved in regulation gradually decreased as the heat stress temperature increased. The regulation process also involved the regulation of immune, endocrine, and nervous systems, including inflammatory bowel disease, the intestinal immune network producing IgA, differentiation of Th1 and Th2 cells, the IL-17 signal transduction pathway, Th17 cell differentiation (immune disease), the AGE-RAGE signal transduction pathway in diabetes complications, etc. The regulation of pathways related to neuroimmune regulation in the body gradually increased with the deepening of heat stress.

## 4. Discussion

Several reports based on cell and animal models have shown that polyphenols have a protective effect on the intestinal barrier; however, research on the regulatory role in heat stress remains limited, and the majority has focused on animal husbandry [13]. A previous study found that different polyphenols, or the same polyphenols under different conditions, interact with each other depending on their mechanism of action and the degree or type of stress being applied [27]. Therefore, the present study involved analyses of the regulatory mechanisms of mung bean polyphenols under different degrees of heat stress. The most important characteristic that distinguishes polyphenols from other natural active substances is their synergistic activity. Previous studies have confirmed that plant polyphenols also have synergistic effects that vary depending on the polyphenol type; however, compared to monomers, their functional activity can be significantly enhanced [28,29]. In the preliminary research of the research group, 57 polyphenolic substances were identified in the mixed polyphenols of mung beans through non-targeted metabolomics analysis. The heat stress marker HSP70 mRNA content confirmed that the regulatory effect of the mixture of vitexin, orientin, and caffeic acid was stronger than that of the mixed mung bean polyphenols. Furthermore, the decrease in HSP70 protein expression at the three heat stress temperatures also confirmed that the three polyphenol mixtures in mung beans had heat stress regulatory ability and that the effect was more obvious at higher temperatures [22].

Minimal research has been conducted on the heat stress regulation effect of mung bean polyphenols. Previous reports have shown that quercetin is a polyphenolic substance in mung beans with a heat stress regulation effect. For example, Cao et al. [30] showed that quercetin and isoquercetin are the main antioxidant components in mung bean soup. After feeding mung bean soup to heat-stress-model rats, it was found that both quercetin and isoquercetin can enter rat plasma, and the levels of malondialdehyde, lactate dehydrogenase, and nitric oxide synthase in the rat plasma before and after heat stress were significantly reduced. The total antioxidant capacity and glutathione levels were also significantly increased, indicating that mung bean soup is more effective than water in preventing heat stress damage. Wang et al. [21] orally fed mung bean soup to heat-stressed mice and found that the HSP70 protein, glutathione, and immunoglobulin G (IgG) contents in the mouse blood were significantly increased. Correlation analysis showed that the increase in these indicators was highly correlated with the flavonoid and polyphenol components in mung beans, indicating that mung bean polyphenols have a regulatory effect on heat stress; however, the regulatory mechanism remains unclear. Bhardwaj et al. found that pre-treatment of A549 cells with quercetin significantly increased cell viability after heat stress, activated endoplasmic reticulum stress-induced autophagy, and activated the IRE1-JNK pathway to increase HSP90, GRP78, and IRE1-α expression. GRP78 expression can decrease PERK, caspase-3, and caspase-4 expression. An increase in GRP78 expression can lead to caspase-4 inactivation and reduce cell apoptosis caused by endoplasmic reticulum stress [31]. The results of the present study also confirmed that mung bean polyphenols have heat stress regulation effects; however, unlike other studies, it was found that orientin and caffeic acid were also key heat stress regulation components in mung beans.

The mechanism of polyphenol heat stress regulation was partially reported and shown to improve signal pathway expression and regulate heat shock proteins, oxidative stress levels, and inflammatory factor levels to exert activity. Furthermore, it was found that different polyphenol regulation mechanisms differed. Tea polyphenols can be downregulated by downregulating HSP70, LDH, CK, CK-MB, and TNF-α. The upregulation of Total Antioxidant Capacity (T-AOC), glutathione peroxidase (GSH-PX), superoxide dismutase (SOD), and nuclear factor erythroid 2-related factor (Nrf2) expression effectively alleviates the effects of heat stress on cells, with the related pathways being Keap1-Nrf2-ARE and AMPK signalling pathways14. Resveratrol can activate the SIRT1-Nrf1/Nrf2 signalling pathway, increase intestinal ATP levels and SOD and catalase (CAT) antioxidant enzyme activity, and inhibit NF-κ. The activation of the B/NLRP3 signalling pathway reduces the expression of inflammatory factors and NLRP3, caspase-1, and p20 proteins, ultimately alleviating heat stress-induced oxidative stress and inflammatory damage. Chlorogenic acid inhibits TLR4/NF-ĸ. The B signalling pathway activates the Nrf2/HO-1 signalling pathway, reduces the MDA content in the jejunum and duodenum, enhances CAT and GSH-PX activity, and relieves intestinal damage in pigs through the expression of antioxidant factors [32]. Ferulic acid can inhibit reactive oxygen species (ROS), Malondialdehyde (MDA), and the nitric oxide (NO) content, enhance SOD activity, and activate the Phosphatidylinositol-4,5-bisphosphate3-kinase/Angry King Team (PI3K/Akt)-mediated Nrf2/HO-1 signalling pathway to alleviate heat stress-induced damage to IEC-6 cells. It can also reduce a series of heat stress-induced injuries by activating the EGFR/Elk signalling pathway and inhibiting MAPK and NF-κB [33], and it reduces the content of inflammatory factors and tight junction protein expression [34]. Curcumin can alleviate heat stress-induced excessive ROS production, cell apoptosis, and mitochondrial damage while maintaining the morphology and function of mitochondria [35]. Curcumin can also reduce the expression of ROS and MDA in cells, enhance antioxidant enzyme activity, and participate in alleviating oxidative stress caused by heat stress by promoting Nrf2, Jnk, Erk, and p38 expression in the MAPK-Nrf2 signalling pathway [36]. Adding curcumin to feed can alleviate physiological disorders caused by heat stress in mice, downregulate cTn-I and Ang-II expression, upregulate GRP78 protein expression, and increase the content of antioxidant indicators such as SOD, CAT, and GSH-Px [16]. In summary, the regulatory mechanism of polyphenolic heat stress is often related to oxidative stress and inflammation-related pathways.

In this study, it was found that the environment-related signalling pathways regulated by mung bean polyphenols included the FoxO, Rap1, and PI3K-Akt signalling pathways. The Rap1 signalling pathway exists in many important cellular processes, such as information and control of cell adhesion and connectivity, cell migration, polarisation, proliferation, and survival [37]. The FoxO signalling pathway is a protein signalling pathway that participates in regulating cell responses to environmental signals. The PI3K-Akt and MAPK signalling pathways are upstream signalling pathways and are ultimately involved in regulating the cell cycle, apoptosis, antioxidant stress, DNA repair, metabolism, etc. [38]. It also involves pathways such as inflammation and immunity, such as IBD; the intestinal immune network that produces IgA; differentiation of Th1, Th2 and Th17 cells; and the IL-17 and AGE-RAGE signalling pathways. This indicated that mung bean polyphenols may exert heat stress protection by participating in protein signalling pathway regulation and thus intervening in cellular processes and immune regulation. From the analysis of the association between metabolomics and transcriptomics, differential metabolites and genes were screened, and the regulatory targets of mung bean polyphenols under different degrees of heat stress differed The expression of the key gene Fos in the regulation of heat stress at 39 °C was involved in the regulation of cell apoptosis, and the Fos protein plays a crucial role in regulating cell growth and metabolism [39]. The key differential metabolic markers were lipids and their derivatives. Among the pathways with simultaneous changes in other selected gene and metabolic sets, amino acid substances constituted the majority of differential metabolites, and most of the differential metabolites were upregulated in content. Lipid metabolism can promote cell apoptosis by regulating membrane permeability and activating different enzymes. Changing lipid-related signalling substances can overcome cell death mechanisms and promote lipid breakdown and synthesis metabolism to meet energy needs and oxidative stress protection [40]. Amino acids are mainly used for the growth and development of tissues and also serve as metabolic fuels to provide energy through liver gluconeogenesis [41]. Glutamate, glutamine, arginine, and leucine improve the inflammatory state of the intestine by preventing villous atrophy and improving the synthesis of tight junction proteins and intestinal barrier function. 3-methylhistidine, creatinine, etc., are biomarkers for the increase in systemic proteolysis in animals caused by heat stress to supply amino acids to maintain cellular function [42]. At 39 °C, mung bean polyphenols mainly regulated heat stress damage by inhibiting cell apoptosis and promoting lipid and amino acid accumulation.

During the heat stress regulation process of mung bean polyphenols at 41 °C, the key genes Hspa1l, Hspa1b, and EHspa1b (which are all members of the heat shock protein 70 family) were induced under stress conditions and had multiple cellular protective effects. They jointly regulated each other, including protein complexes used for antigen presentation, stabilising intracellular proteins, and promoting protein folding. The HSP70 gene exhibits polymorphism at the Hspa1b and HSPA1l loci [43], among which Hspa1l plays an important role in regulating IBD [44]. Regulation by mung bean polyphenols downregulated Hspa1b and EHspa1b caused by heat stress. Both Cox8a and NDUFA4L2 are involved in electron transfer within mitochondria. Cox8a achieves transmembrane transfer of cytochrome c to oxygen and protons, which is related to membrane integrity [45]. Cox8a can also optimise adaptive evolutionary changes in aerobic energy metabolism in the respiratory chain mechanism [46]. NDUFA4L2 is HIF-1 localised in mitochondria α target genes. Previous studies have shown that mitochondrial NDUFA4L2 can weaken oxidative stress-induced apoptosis in nucleus pulposus cells by inhibiting mitochondrial autophagy [47], and the overexpression of NDUFA4L2 is associated with poor prognosis in colorectal cancer patients [48]. The main effect of Pde6a (downregulation) is to increase the intracellular calcium ion concentration, which is beneficial for calcium ion influx and improves cell function. Grin1 and Tymp are signal transduction-related factors between neurons, and abnormal transcriptional regulation of Grin1 may be related to the course of neurological and psychiatric disorders [49]. Grin1 can also regulate immune system activity in other cell types, affect cell proliferation and differentiation, and participate in various biological processes, such as regulating inflammatory responses. The Tymp gene can encode thymidine phosphorylase, and its mutations can cause mitochondrial neurogastrointestinal neuromuscular disorders. Tymp deficiency can lead to mitochondrial dysfunction and broader metabolic disorders [50]. Jund is a member of the transcription factor family that activates protein 1, regulating cell differentiation, proliferation, and apoptosis [51] and protecting cells from oxidative stress by limiting ROS production [52]. ENTPD3, a membrane-bound enzyme that can hydrolyse extracellular ATP and ADP, is closely related to energy metabolism and is mainly expressed throughout the central nervous system [53]. There are currently few reports on the key genes mentioned above in heat stress regulation. At 41 °C, mung bean polyphenols attenuated heat stress-induced cell damage or apoptosis by regulating heat shock proteins and inhibiting mitochondrial function and some neurodisease-related genes.

The key regulatory gene Gria4 in heat stress regulation by mung bean polyphenols at 43 °C is a glutamate receptor, which is the main excitatory neurotransmitter receptor in the mammalian brain. The Gria4 gene is related to the neuronal system and the neural active ligand–receptor interaction function of heat tolerance, and it is a potential anti-heat stress regulatory gene [54]. The mt-Nd3 and mt-Nd4l genes predict the activation of NADH dehydrogenase (ubiquinone) activity in mitochondria, which is associated with mitochondrial function and regulation of neuropathy [55]. PE (16:1 [9Z]/15:0), PC (14:0/14:0), and PGH23 metabolic markers are phosphatidylcholine-like substances that have the function of repairing brain cells. The impact of heat stress on the body is essentially associated with complex neuroimmune regulation, which can be achieved through the microbiota gut–brain axis to regulate the brain system. Neurotransmitters, neuropeptides, growth factors, neuroendocrine hormones, and cytokines are common regulatory substances in complex systems [56]. The key genes and metabolites identified in the present study indicated that mung bean polyphenols alleviated heat stress damage to intestinal cells by regulating nerve-related genes under severe heat stress at 43 °C.

In this study, integrated analysis of metabolomics and transcriptomics was performed to investigate the effects of mung bean polyphenols on gene expression and metabolite levels in intestinal cells under different heat stress temperatures. Through the mining of key genes, pathways, and metabolites, the mechanism of heat stress-regulating activity of mung bean polyphenols was preliminarily analysed. However, the key regulatory targets and the upstream and downstream regulatory relationships of each key pathway obtained in this study need to be further confirmed by Western blot and other methods. At the same time, this study divided different heat stress degrees by a temperature gradient of 2 °C, but as a dietary heat stress intervention method, the discovery of mung bean polyphenols on the regulation law of different heat stress degrees is also necessary, and in-depth research on the regulation mechanism of heat stress at 38 °C and 40 °C is also the next step.

## 5. Conclusions

This study confirmed that mung bean polyphenols had the ability to regulate heat stress by their ability to regulate at the level of heat shock proteins. Eight key metabolic markers (Phe Gly, inosine 2-phosphate, 2-propylthiazolidine-4-carboxylic acid, S-allyl-L-cysteine, alanine tryptophan, creatine, L-carnitine, and phosphocreatine) were found to be involved in the heat stress regulation of mung bean polyphenols. Choline metabolism, lipid metabolism, carbohydrate biosynthesis and metabolism, and nervous system pathways were the key metabolic regulatory pathways. From a genetic perspective, the key regulatory genes and metabolic pathways differed under different heat stress temperatures. The main heat stress regulation pathway of mung bean polyphenols at 39 °C was choline metabolism in cancer, that at 41 °C was pyrimidine metabolism, and that at 43 °C was the retrograde endorphin signalling pathway. The FoxO, Rap1, and PI3K Akt signalling pathways were key environmental-related regulatory signalling pathways. At 39 °C, mung bean polyphenols mainly regulated heat stress injury by inhibiting cell apoptosis and promoting lipid and amino acid accumulation. At 41 °C, mung bean polyphenols mainly attenuated heat stress-induced cell damage or apoptosis by regulating heat shock proteins and inhibiting mitochondrial function and some neurodisease-related genes. Severe heat stress at 43 °C mainly alleviated heat stress damage to intestinal cells by regulating nerve-related genes.

## Figures and Tables

**Table 1:** Key pathways of heat stress regulation by mung bean polyphenols.

Number	Primary Pathway	Secondary Pathway	Path Description	Quantity of Differential Metabolites	Metabolite Name
1	Human diseases	Cancer: Overview	Choline metabolism in cancer	20	Cytidine, choline phosphate, citrine, PC(18:3(6Z,9Z,12Z)/P-16:0), PC(18:3(9Z,12Z,15Z)/20:0), LysoPC(16:0/0:0), PC(14:0/14:0), GPCho(18:1/16:0), PC(18:1(9Z)/P-16:0), LysoPC(20:4(8Z,11Z,14Z,17Z)/0:0), PC(16:0/18:2(9Z,12Z)), PC(16:1(9Z)/P-18:0), PC(16:0/16:1(9Z)), PC(14:0/16:0), GPCho(14:0/18:1), PC(18:1(11Z)/14:1(9Z)), PC(16:1(9Z)/14:0), PC(18:0/0:0), PC(18:0/18:3(9Z,12Z,15Z)), LysoPC(18:0/0:0)
2	Metabolise	Lipid metabolism	Glycerophospholipid metabolism	28	Cytidine, choline phosphate, citrine, LysoPC(16:0/0:0), PC(18:3(9Z,12Z,15Z)/20:0), PC(14:0/14:0), PC(18:3(6Z,9Z,12Z)/P-16:0), PE(18:1(11Z)/18:0), GPCho(18:1/16:0), PC(18:1(9Z)/P-16:0), PE-NMe2(18:1(9Z)/16:1(9Z)), LysoPC(20:4(8Z,11Z,14Z,17Z)/0:0), PC(16:0/18:2(9Z,12Z)), PE(18:1(9Z)/18:0), PC(16:1(9Z)/P-18:0), PE(16:0/18:0), PC(16:0/16:1(9Z)), PE(16:1(9Z)/P-18:1(9Z)), PC(14:0/16:0), GPCho(14:0/18:1), PC(18:1(11Z)/14:1(9Z)), PC(16:1(9Z)/14:0), PC(18:0/0:0), PE(18:3(6Z,9Z,12Z)/P-18:1(9Z)), PC(18:0/18:3(9Z,12Z,15Z)), LysoPC(18:0/0:0), PS(18:1(9Z)/18:0), PE(18:1(11Z)/18:1(11Z))
3	Body system	Nervous system	Retrograde endogenous cannabinoid signalling	22	L-Glutamate, PC(14:0/14:0), PE(18:1(11Z)/18:0), GPCho(18:1/16:0), PC(18:1(9Z)/P-16:0), PC(16:0/18:2(9Z,12Z)), PC(16:1(9Z)/P-18:0), PC(16:0/16:1(9Z)), PC(14:0/16:0), GPCho(14:0/18:1), PC(18:1(11Z)/14:1(9Z)), PC(16:1(9Z)/14:0), PE(18:3(6Z,9Z,12Z)/P-18:1(9Z)), PC(18:0/18:3(9Z,12Z,15Z)), PE(18:1(11Z)/18:1(11Z)), PC(18:3(9Z,12Z,15Z)/20:0), PC(18:3(6Z,9Z,12Z)/P-16:0), PE(18:1(9Z)/18:0), PE(16:0/18:0), PE(16:1(9Z)/P-18:1(9Z)), PE(16:1(9Z)/15:0), PGH2
4	Metabolism	Biosynthesis and metabolism of sugars	Glycosylphosphatidylinositol (GPI)—anchor biosynthesis	9	Urea diphosphate *N*-acetylglucosamine PE(18:0/18:1(11Z)), PE(18:1(9Z)/18:0), PE(16:0/18:0), PE(16:1(9Z)/P-18:1(9Z)), PE(18:1(11Z)/18:0), PE(18:3(6Z,9Z,12Z)/P-18:1(9Z)), PE(18:1(11Z)/18:1(11Z)), PE(16:1(9Z)/15:1)

**Table 2:** Differential metabolite expression patterns of choline metabolic pathways in mung bean polyphenols 39 °C heat stress-regulated.

Metabolite Name	Number	KEGG ID	Mass to Charge Ratio	Difference Multiple	Expression Situation
Choline Phosphate	metab_428	C00588	184.07	1.0361	up
LysoPC(16:0/0:0)	metab_430	C04230	496.34	1.0341	up
PC(14:0/14:0)	metab_441	C00157	678.51	1.0816	up
GPCho(18:1/16:0)	metab_664	C00157	782.57	0.9298	down
PC(18:1(9Z)/P-16:0)	metab_671	C00157	766.57	0.9453	down
LysoPC(20:4(8Z,11Z,14Z,17Z)/0:0)	metab_1059	C04230	544.34	1.029	up
PC(16:0/18:2(9Z,12Z))	metab_1086	C00157	780.55	0.9643	down
PC(16:1(9Z)/P-18:0)	metab_1098	C00157	766.57	1.0728	up
PC(16:0/16:1(9Z))	metab_1118	C00157	732.55	0.9215	down
PC(14:0/16:0)	metab_1260	C00157	728.52	1.1231	up
GPCho(14:0/18:1)	metab_1291	C00157	754.54	1.0591	up
PC(18:1(11Z)/14:1(9Z))	metab_1488	C00157	730.54	1.1358	up
PC(16:1(9Z)/14:0)	metab_1492	C00157	704.53	1.1953	up
PC(18:0/0:0)	metab_1493	C04230	546.35	1.6141	up
PC(18:0/18:3(9Z,12Z,15Z))	metab_3404	C00157	784.58	1.0545	up
LysoPC(18:0/0:0)	metab_3511	C04230; C04317	524.37	1.0416	up

**Table 3:** Mung bean polyphenols at 41 °C with the Mode-k cell heat stress-regulated metabolic set and gene set KEGG pathway enrichment.

Channel Name	Classification of Primary Pathways	Secondary Pathway Classification	Genetic Changes
Purine metabolism	Metabolism	Nucleotide metabolism	Entpd3 significantly upregulated 26.0902-fold; Pde6a significantly downregulated 0.0549-fold
Huntington’s disease	Human diseases	Neurodegenerative diseases	Grin1 significantly downregulated 0.04480-fold; Cox8a downregulated 0.4492-fold; Ndufa4l2 downregulated 0.1631-fold
Toxoplasmosis	Human diseases	Infectious diseases: parasitic diseases	Hspa1b downregulated 0.3346-fold; Hspa1l significantly upregulated 21.4120-fold
Oestrogen signalling pathway	Body system	Endocrine system	Hspa1b downregulated 0.3346-fold; Hspa1l significantly upregulated 21.4120-fold
Spliceosome	Genetic information processing	Transcription	Hspa1b downregulated 0.3346-fold; Hspa1l significantly upregulated 21.4120-fold
Antigen processing and presentation	Body system	Immune system	Hspa1b downregulated 0.3346-fold; Hspa1l significantly upregulated 21.4120-fold
Measles	Human diseases	Infectious diseases: viral	EHspa1bb downregulated 0.3346-fold; Hspa1l significantly upregulated 21.4120-fold
Oxidative phosphorylation	Metabolism	Energy metabolism	Cox8ab downregulated 0.4492-fold; Ndufa4l2b downregulated 0.1631-fold
Myocardial contractions	Organic systems	Circulatory system	Cox8a downregulated 0.4492-fold; Cacnb4 upregulated 2.1476-fold
Pyrimidine metabolism	Metabolism	Nucleotide metabolism	Tymp significantly downregulated 0.0185-fold; Entpd3 significantly upregulated 26.0902-fold
MAPK signalling pathway	Environmental information processing	Signal transduction	Hspa1b downregulated 0.3346-fold; Cacnb4 upregulated 2.1476-fold; Jund downregulated 0.4006-fold; Hspa1l significantly upregulated 21.4120-fold
Longevity regulation pathways—multiple species	Body system	Ageing	Hspa1b downregulated 0.3346-fold; Hspa1l upregulated 21.4120-fold
Legion disease	Human diseases	Infectious diseases: bacteria	Hspa1b downregulated 0.3346-fold; Hspa1l upregulated 21.4120-fold
Prion disease	Human diseases	Neurodegenerative diseases	Hspa1b downregulated 0.3346-fold; Cox8a downregulated 0.4492-fold; Ndufa4l2 downregulated 0.1631-fold; Hspa1l significantly upregulated 21.4120-fold

**Table 4:** Expression patterns of differential metabolites under 43 ℃ heat stress in the retrograde endorphin signaling pathway.

Metabolite	Name	KEGG ID	Mass-to-Charge Ratio	Difference Multiple	Expression Situation
PE(16:1(9Z)/15:0)	metab_619	C00350	708.51	1.0896	up
PC(14:0/14:0)	metab_441	C00157	678.51	1.0812	up
PGH2	metab_4048	C00427	351.22	1.0264	up

**Table 5:** Correlation analysis of differential genes and metabolites.

Gene Name	Correlation	PC(14:0/14:0)	PE(16:1(9Z)/15:0)	PGH2
ENSMUSG00000064360(mt-Nd3)	negative correlation	**	-	*
ENSMUSG00000025892(Gria4)	positive correlation	-	*	**

Note: * *p* < 0.05; ** *p* < 0.01.

## Data Availability

The main data supporting the results of this study are available within this paper and its Supplementary Materials.

## Supplementary Materials

The following supporting information can be downloaded at https://www.mdpi.com/article/10.3390/nu17010088/s1: Table S1 List of differential metabolites in the 39 °C HS-39 °C DF group of MODE-K cells. Table S2 List of differential metabolites in the 41 °C HS-41 °C DF group of MODE-K cells. Table S3 List of differential metabolites in the 43 °C HS-43 °C DF group of MODE-K cells. Table S4 Results of significant pathway enrichment analysis for the 39 °C HS-39 °C DF group. Table S5 Results of significant pathway enrichment analysis for the 41 °C HS-41 °C DF group. Table S6 Results of significant pathway enrichment analysis for the 43 °C HS-43 °C DF group. Table S7 List of key genes in the regulation of heat stress with mung bean polyphenols at 39 °C. Table S8 List of key genes in the regulation of heat stress with mung bean polyphenols at 41 °C. Table S9 List of key genes in the regulation of heat stress with mung bean polyphenols at 43 °C. Table S10 Table of enrichment information for the 39 °C HS-39 °C DF group KEGG pathway. Table S11 Table of enrichment information for the 41 °C HS-41 °C DF group KEGG pathway. Table S12 Table of enrichment information for the 43 °C HS-43 °C DF group KEGG pathway. Table S13 Enrichment statistics of gene set and metabolic set KEGG pathway regulated by heat stress in mung bean polyphenols 39 °C Mode-k cells. Table S14 Enrichment statistics of gene set and metabolic set KEGG pathway regulated by heat stress in mung bean polyphenols 41 °C Mode-k cells. Table S15 Enrichment statistics of gene set and metabolic set KEGG pathway regulated by heat stress in mung bean polyphenols 43 °C Mode-k cells.
