# Supplementary material for: Integrating Metabolomics and Transcriptomics to Analyse and Reveal the Regulatory Mechanisms of Mung Bean Polyphenols on Intestinal Cell Damage Under Different Heat Stress Temperatures
Source: Nutrients. 2024 Dec 29;17(1):88. doi: 10.3390/nu17010088 (PMC11722878; doi:10.3390/nu17010088)
Supplement: Supplementary file 1 [file nutrients-17-00088-s001.zip › nutrients-3332331-supplementary.pdf]

Table S1 List of differential metabolites in the 39 ℃ HS-39 ℃ DF group of MODE-K cells

Table S2 List of differential metabolites in the 41 ℃ HS-41 ℃ DF group of MODE-K cells

Table S3 List of differential metabolites in the 43 ℃ HS-43 ℃ DF group of MODE-K cells

Table S4 Results of significant pathway enrichment analysis for the 39 ℃ HS-39 ℃ DF group

Table S5 Results of significant pathway enrichment analysis for the 41 ℃ HS-41 ℃ DF group

Table S6 Results of significant pathway enrichment analysis for the 43 ℃ HS-43 ℃ DF group

Table S7 List of key genes in the regulation of heat stress with mung bean polyphenols at 39°C

Table S8 List of key genes in the regulation of heat stress with mung bean polyphenols at 41°C

Table S9 List of key genes in the regulation of heat stress with mung bean polyphenols at 43°C

Table S10 Table of enrichment information for the 39 °C HS-39 °C DF group KEGG pathway

Table S11 Table of enrichment information for the 41 °C HS-41 °C DF group KEGG pathway

Table S12 Table of enrichment information for the 43 °C HS-43 °C DF group KEGG pathway

Table S13 Enrichment statistics of gene set and metabolic set KEGG pathway regulated by heat stress in mung bean polyphenols 39°C Mode-k cells

Table S14 Enrichment statistics of gene set and metabolic set KEGG pathway regulated by heat stress in mung bean polyphenols 41°C Mode-k cells

Table S15 Enrichment statistics of gene set and metabolic set KEGG pathway regulated by heat stress in mung bean polyphenols 43°C Mode-k cells

Table S1 List of differential metabolites in the 39 ℃ HS-39 ℃ DF group of MODE-K cells

Number	Metabolite name	Content changes	M/Z	RT	VIP	FC(HS39/CON)	P_value
1	SM(d18:1/16:0)	down	703.57	6.8104	1.33	0.98	0.0494
2	SM(d17:1/18:1(12Z)-O(9S,10R))	up	761.59	5.8728	1.90	1.05	0.0026
3	Aspartame	down	295.13	2.8669	2.30	0.92	0.0045
4	Hypoglycin B	down	334.14	3.2814	2.71	0.86	0.0071
5	DG(10:0/0:0/20:5(6E,8Z,11Z,14Z,17Z)-OH(5))	up	285.19	3.297	1.58	1.05	0.0311
6	ROSAMICIN	down	564.36	3.4922	2.87	0.85	0.0435
7	Choline Phosphate	up	184.07	5.8415	1.55	1.04	0.0001
8	LysoPC(16:0/0:0)	up	496.34	5.8415	1.56	1.03	0.0064
9	FAHFA(18:0/8-O-18:0)	down	584.56	5.8571	1.16	0.98	0.0019
10	Cer(d18:0/16:0)	down	540.53	5.865	1.40	0.97	0.0092
11	PC(14:0/14:0)	up	678.51	5.865	2.17	1.08	0.0041
12	(2S)-2-Amino-6-[(3-formylpiperidin-1-yl)amino]hexanoic acid	down	532.38	5.8963	1.49	0.96	0.0448
13	PE(18:1(11Z)/18:0)	down	746.57	5.9511	2.97	0.89	0.0020
14	2-(Octadecyloxy)ethanol	up	359.29	6.3101	1.33	1.03	0.0500
15	GPCho(18:1/16:0)	down	782.57	6.6226	2.39	0.93	0.0127
16	PC(18:1(9Z)/P-16:0)	down	766.57	6.7634	1.90	0.95	0.0421
17	Pantothenic Acid	down	220.12	2.0701	1.26	0.98	0.0398
18	LysoPC(20:4(8Z,11Z,14Z,17Z)/0:0)	up	544.34	7.2478	1.29	1.03	0.0471
19	Amastatin	up	516.31	7.2478	1.24	1.03	0.0463
20	PC(16:0/18:2(9Z,12Z))	down	780.55	7.2321	1.77	0.96	0.0006
21	PC(16:1(9Z)/P-18:0)	up	766.57	7.2244	2.25	1.07	0.0003
22	PC(16:0/16:1(9Z))	down	732.55	7.2088	2.68	0.92	0.0000
23	17-N,N-Diethylcarbamoyl-4-methyl-4-azaandrostane-3-one	up	430.35	6.2788	1.28	1.03	0.0414
24	PC(14:0/16:0)	up	728.52	6.271	2.61	1.12	0.0218
25	GPCho(14:0/18:1)	up	754.54	6.1695	1.99	1.06	0.0246
26	Aglepristone	up	464.31	6.1072	1.28	1.03	0.0417
27	Solasodine	up	378.32	6.0839	3.51	1.28	0.0116
28	Corosin	down	551.36	6.0057	1.45	0.96	0.0307
29	LysoPA(16:0/0:0)	down	452.28	5.9589	1.13	0.98	0.0168
30	SM(d18:0/16:1(9Z))	up	725.56	5.8963	3.94	1.32	0.0027
31	Ubiquinone-8	up	744.59	5.8806	2.45	1.08	0.0008

32	PC(18:1(11Z)/14:1(9Z))	up	730.54	5.8728	3.03	1.14	0.0001
33	PC(16:1(9Z)/14:0)	up	704.52	5.865	3.51	1.20	0.0000
34	PC(18:0/0:0)	up	546.35	5.865	4.87	1.61	0.0083
35	Nuatigenin	up	494.32	5.8415	1.45	1.03	0.0004
36	Madlongiside C	down	650.40	4.3587	3.52	0.78	0.0456
37	Chelidonine	up	336.12	4.3432	2.77	1.15	0.0001
38	10-Hydroxycarbazepine	up	255.11	3.8357	3.29	1.25	0.0002
39	Simonin IV	down	672.41	4.3978	3.62	0.77	0.0462
40	3-Hydroxy-5,5,8a-Trimethyl-3,4,4a,6,7,8-Hexahydronaphthalene-2-Carboxylic Acid	down	261.14	2.7419	1.92	0.93	0.0410
41	Pro Ile	up	229.15	2.2817	1.66	1.05	0.0041
42	N-Methylisoleucine	down	146.12	2.2505	1.36	0.96	0.0309
43	Vomifoliol	up	247.13	2.2271	1.68	1.05	0.0230
44	Gamma-Glutamyltyrosine	down	311.12	2.0389	2.10	0.92	0.0167
45	Phe Gly	up	223.11	2.0233	3.03	1.18	0.0040
46	Phe Ser	up	253.12	1.7819	2.49	1.18	0.0435
47	Pro-Pro-Pro	up	310.18	1.774	1.68	1.04	0.0025
48	Tyr Gly	up	239.10	1.7584	1.65	1.05	0.0017
49	Adenosine 3',5'-Diphosphate	down	428.04	1.3052	1.46	0.96	0.0413
50	Xanthosine 5'-Monophosphate	down	365.05	1.2114	2.04	0.89	0.0257
51	4-Guanidinobutanoic Acid	down	146.09	0.9607	2.59	0.82	0.0331
52	Uridine diphosphate glucose	down	630.07	0.7802	1.93	0.94	0.0042
53	Uridine diphosphate-N-acetylgalactosamine	down	652.05	0.7724	1.58	0.96	0.0274
54	Imidazole Lactic Acid	down	157.06	0.6005	1.16	0.97	0.0293
55	Creatine	down	132.08	0.5926	1.12	0.98	0.0488
56	N,N'-Diethylthiourea	down	133.08	0.5926	1.21	0.97	0.0448
57	L-Carnitine	down	162.11	0.5694	1.11	0.98	0.0274
58	Theophylline	down	219.03	0.5383	1.32	0.97	0.0285
59	PC(O-14:0/16:1(9Z))	down	690.54	5.9823	3.72	0.81	0.0001
60	PE(18:3(6Z,9Z,12Z)/P-18:1(9Z))	down	756.55	6.8026	2.58	0.92	0.0034
61	PC(18:3(9,11,15)-OH(13)/16:0)	down	754.53	7.2321	2.51	0.93	0.0000
62	PC(18:1/0:0)	up	522.36	5.8258	1.57	1.03	0.0021
63	1-Ethyl-3-(dimethyl-aminopropyl)-carbodiimide	down	188.18	0.5383	1.28	0.97	0.0321
64	Dimethyl Sulfoxide	up	79.02	0.6629	6.02	1.95	0.0000
65	Uridine diphosphategalactose	down	608.09	1.3364	1.91	0.95	0.0039

66	PC(18:0/18:3(9Z,12Z,15Z))	up	784.58	5.8963	2.06	1.05	0.0014
67	Homomethionine	down	164.07	1.8053	1.96	0.92	0.0041
68	Histidylglycine	down	195.09	1.8366	1.29	0.97	0.0113
69	Valylhydroxyproline	up	195.11	2.2505	1.54	1.05	0.0105
70	S-Butylcysteine sulfoxide	up	176.07	2.2973	1.77	1.09	0.0319
71	Glucose-uridine-C1,5'-diphosphate	down	630.07	1.2739	1.60	0.96	0.0208
72	LysoPC(18:0/0:0)	up	524.37	5.8493	1.75	1.04	0.0107
73	Griseolic acid	down	414.04	1.0631	2.11	0.90	0.0177
74	Citric Acid	down	191.02	1.1511	1.99	0.92	0.0015
75	ADP Ribose	down	540.05	1.3168	1.57	0.95	0.0462
76	NAD+	down	662.10	1.3248	1.80	0.93	0.0460
77	Glutamylphenylalanine	down	293.11	3.4238	2.30	0.90	0.0071
78	Gabazine	down	332.13	3.8309	2.61	0.86	0.0033
79	Indolelactic acid	down	204.07	4.705	1.34	0.96	0.0402
80	Apigenin	up	269.05	5.6624	4.14	1.49	0.0000
81	PE(16:1/0:0)	down	450.26	5.9076	1.00	0.98	0.0039
82	PE(16:0/0:0)	down	452.28	5.9859	1.21	0.97	0.0235
83	Magnolol	up	265.12	6.3044	5.52	1.95	0.0002
84	2-Arachidonyl Glycerol ether	up	401.25	6.3044	5.10	1.77	0.0028
85	PS(18:1(9Z)/18:0)	down	810.53	6.6384	1.56	0.95	0.0500
86	PS(18:1(9Z)/0:0)	up	522.28	7.1148	1.01	1.02	0.0147
87	UDP-N-acetyl-D-mannosamine	down	606.07	0.7668	1.54	0.97	0.0094
88	Cer(d18:1/24:1(15Z))	down	692.62	6.8998	1.95	0.92	0.0115
89	ADP	down	426.02	0.9187	1.71	0.95	0.0268
90	Uridine 5'-diphosphoglucuronic acid	down	579.03	0.9346	1.41	0.97	0.0256
91	Cerebroside B	down	748.53	6.4603	1.28	0.97	0.0230
92	PE(18:0/0:0)	down	480.31	6.0964	1.47	0.97	0.0014
93	PE(18:1(9Z)/0:0)	down	478.29	5.9859	1.12	0.98	0.0023
94	XMP	down	363.04	1.1991	3.54	0.75	0.0250
95	Uridine diphosphate-N-acetylglucosamine	down	606.07	0.5902	1.74	0.96	0.0013
96	Gamma-Glu-leu	down	259.13	3.2804	1.76	0.93	0.0375
97	L-Tyrosine	down	180.07	1.6623	1.00	0.98	0.0488
98	GDP-Beta-L-Fucose	down	588.07	1.0951	2.55	0.86	0.0158
99	Cyanidin 3-O-alpha-L-arabinoside	down	464.10	5.6624	1.48	0.96	0.0063
100	Epirubicin	up	588.17	6.0964	1.09	1.02	0.0177
101	PE(18:1(11Z)/18:1(11Z))	down	788.55	7.1148	1.06	0.98	0.0328
102	Taurine	down	124.01	0.5504	1.26	0.97	0.0209

103	Fructosamine	down	214.05	0.5504	1.34	0.97	0.0139
104	L-Glutamate	down	146.05	0.5662	1.11	0.98	0.0480
105	Phenylacetylglutamine	down	309.11	2.5872	2.37	0.89	0.0036

Note: RT： retention time; m/z: mass to core ratio; VIP：The variable projection importance of the first principal component in the OPLS-DA model is an indicator for screening differential metabolites; FC(HS39/CON)：39 °C HS-CON group differential metabolite change multiple . P_value：The P-value of Student's t-test.

Table S2 List of differential metabolites in the 41 °C HS-41 °C DF group of MODE-K cells

Number	Metabolite name	Content changes	M/Z	RT	VIP	FC(HS39/CON)	P_value
1	SM(d18:1/16:0)	down	703.57	6.8104	1.27	0.98	0.0308
2	Tranexamic Acid	up	158.12	2.8045	1.11	1.02	0.0206
3	DG(10:0/0:0/20:5(6E,8Z,11Z,14Z,17Z)-OH(5))	up	285.19	3.297	1.61	1.04	0.0078
4	Santene hydrate	up	123.12	3.9764	1.51	1.05	0.0433
5	Hispiduloside	up	463.12	4.2495	4.71	1.50	0.0000
6	Penicilloic acid	up	353.12	4.5537	2.18	1.07	0.0022
7	Cis-Dihydrocarvone	up	170.15	5.4361	1.69	1.07	0.0333
8	Nordihydrocapsaicin	up	294.20	5.639	1.08	1.02	0.0234
9	D-Xylono-1,5-lactone	up	297.08	5.8258	1.83	1.05	0.0010
10	FAHFA(18:0/8-O-18:0)	down	584.56	5.8571	1.22	0.98	0.0313
11	PC(14:0/14:0)	up	678.51	5.865	2.14	1.09	0.0250
12	2-Aminopyridine	down	95.06	7.5214	1.03	0.98	0.0201
13	5-Hydroxy-L-tryptophan	up	221.09	1.774	2.19	1.11	0.0076
14	Corosin	up	551.36	6.0057	1.02	1.02	0.0254
15	(+/-)-Pelletierine	up	142.12	6.0057	1.21	1.03	0.0152
16	4-Vinylcyclohexene	up	109.10	5.9511	1.71	1.05	0.0101
17	Dihomolinoleic acid	up	298.27	5.9432	1.40	1.03	0.0089
18	Alpha-Terpineol acetate	up	214.18	5.9432	1.71	1.05	0.0114
19	Indoleacrylic acid	up	188.07	2.4151	1.17	1.02	0.0488
20	Dihomo-gamma-linolenic acid	up	339.29	5.8728	1.52	1.04	0.0006
21	22-Hydroxydocosanoic acid	up	374.36	5.8102	2.72	1.13	0.0186
22	Polyoxyethylene 40 monostearate	up	346.33	5.787	2.65	1.12	0.0127
23	3-ketosphingosine	up	298.27	5.7559	1.22	1.03	0.0283
24	Swainsonine	up	174.11	2.898	2.27	1.09	0.0012
25	(R)-Sulcatol	up	298.27	5.639	1.41	1.04	0.0314

26	Phenolsulfonphthalein	up	355.06	4.7882	2.20	1.08	0.0022
27	Lumichrome	up	243.09	4.6553	2.12	1.09	0.0089
28	Gamma-Glutamylglutamic acid	up	309.13	4.4291	2.26	1.08	0.0057
29	Chelidonine	up	336.12	4.3432	3.54	1.23	0.0000
30	Isovitexin	up	433.11	4.1559	3.16	1.15	0.0001
31	10-Hydroxycarbazepine	up	255.11	3.8357	4.15	1.37	0.0000
32	Luteolin-6-C-Glucoside	up	449.11	3.8279	2.05	1.06	0.0014
33	Etozolin	up	329.09	3.8279	1.53	1.04	0.0473
34	4-Piperidinone, 1-hydroxy-2,2,6,6-tetramethyl-	up	172.13	3.8045	1.56	1.04	0.0415
35	Penicillin G	up	335.11	3.3984	2.06	1.07	0.0035
36	3-Methylcrotonaldehyde	down	85.07	3.3984	1.24	0.97	0.0366
37	Fusaric acid	up	180.10	3.1799	1.79	1.06	0.0143
38	1,5-Naphthalenediamine	up	159.09	2.4073	1.48	1.04	0.0079
39	Pro Ile	up	229.15	2.2817	1.37	1.03	0.0345
40	Vomifoliol	up	247.13	2.2271	1.62	1.05	0.0169
41	Glycyl-leucine	up	189.12	2.1563	2.28	1.12	0.0165
42	Ala-Leu	up	203.14	2.1328	2.23	1.13	0.0428
43	G-Glu-Val	up	247.13	1.93	1.61	1.05	0.0141
44	Phe Ser	up	253.12	1.7819	1.96	1.13	0.0336
45	Val Val	up	217.15	1.774	1.07	1.02	0.0482
46	Tyr Gly	up	239.10	1.7584	1.62	1.06	0.0034
47	9H-Purine-9-ol	up	137.05	1.7584	1.59	1.04	0.0356
48	Cis-Acetylacrylate	down	79.02	1.5629	1.13	0.98	0.0063
49	[(2R,5R)-5-(2-Amino-6-oxo-1H-purin-9-yl)-3,4-dihydroxyoxolan-2-yl]methyl dihydrogen phosphate	up	386.05	1.5161	1.24	1.03	0.0111
50	5-Hydroxymethyl-2-furancarboxaldehyde	up	168.07	1.5004	1.15	1.03	0.0334
51	(5Z,7E)-9,10-Seco-5,7,10(19)-cholestatriene	down	369.35	1.4067	1.33	0.98	0.0005
52	Pyridoxal	up	168.07	1.102	1.59	1.05	0.0021
53	Beta-D-Glucosamine	up	162.08	0.921	1.46	1.04	0.0314
54	Geranyl Acetate	up	214.18	5.6703	1.07	1.02	0.0404
55	2-(2-Methoxyethoxy)ethanol	down	159.04	0.5617	1.12	0.98	0.0116
56	1,6-Hexanediamine	up	158.17	0.5383	2.29	1.09	0.0110
57	Spermidine	up	146.17	0.4834	1.71	1.04	0.0191
58	Heptanoic acid	up	172.13	5.9432	2.33	1.09	0.0175
59	PC(18:3(9Z,12Z,15Z)/20:0)	down	794.60	6.1695	1.66	0.97	0.0021
60	Pregeijerene	down	163.15	6.0525	1.16	0.98	0.0249
61	PC(18:3(6Z,9Z,12Z)/P-16:0)	down	740.55	7.2244	1.81	0.95	0.0112

62	PC(O-16:0/20:4(8Z,11Z,14Z,17Z))	down	768.59	7.2321	1.82	0.95	0.0461
63	PC(O-18:1(11Z)/16:0)	down	746.60	5.8806	1.19	0.98	0.0153
64	Sphinganine	up	302.31	5.6859	1.53	1.04	0.0167
65	1-(1,1-dioxothiolan-3-yl)-5-thiophen-2-ylpyrazole-3-carboxylic acid	down	313.03	0.5539	1.84	0.94	0.0361
66	Cholestan-3-one	down	369.35	0.3974	1.20	0.98	0.0010
67	Dimethyl Sulfoxide	up	79.02	0.6629	6.78	2.27	0.0000
68	3-ketosphinganine	up	300.29	5.6546	1.58	1.04	0.0142
69	8-Amino-7-oxononanoic acid	up	170.12	5.9511	2.64	1.13	0.0266
70	Xanthine	up	153.04	1.5004	1.73	1.05	0.0040
71	Piperidine	up	86.10	1.5708	1.26	1.02	0.0363
72	3-Methoxyanthranilate	up	168.07	1.7819	1.49	1.05	0.0000
73	Asp Ile Glu	up	376.17	2.0155	3.05	1.21	0.0124
74	Dioctyl phthalate	up	413.27	6.0839	1.17	1.02	0.0477
75	Glucosamine	up	162.08	0.6082	1.30	1.03	0.0265
76	Indole-3-Carboxaldehyde	up	146.06	2.4151	1.25	1.03	0.0342
77	2-Hydroxy-3-Methylbutyric Acid	down	117.05	2.2672	1.52	0.96	0.0214
78	L-Phenylalanine	up	164.07	2.2992	1.21	1.03	0.0459
79	Undeca-3,6,9-trienedioylcarnitine	up	374.16	2.5712	2.62	1.20	0.0397
80	Citicoline	up	510.09	4.3082	2.42	1.11	0.0005
81	Chrysophanein	up	461.11	4.6865	4.69	1.53	0.0000
82	Apigenin	up	269.05	5.6624	4.04	1.39	0.0004
83	LysoPE(P-18:1(9Z)/0:0)	up	462.30	6.0334	1.42	1.03	0.0340
84	LysoPE(P-18:0/0:0)	up	464.31	6.1117	1.62	1.05	0.0173
85	N-Arachidonoyl Isoleucine	up	438.30	6.1197	1.70	1.06	0.0485
86	PC(18:1(11Z)/P-16:0)	down	788.58	6.5472	2.32	0.91	0.0336
87	PE(18:0/18:1(11Z))	down	766.54	6.5552	1.94	0.94	0.0482
88	PE-NMe2(18:1(9Z)/16:1(9Z))	down	742.54	6.6325	1.77	0.95	0.0228
89	PS(18:1(9Z)/18:0)	down	810.53	6.6384	1.72	0.95	0.0470
90	PE-NMe(15:0/18:1(9Z))	down	716.52	6.6927	1.79	0.95	0.0262
91	PE(18:1(9Z)/18:0)	down	726.54	6.7993	2.01	0.94	0.0272
92	PE-NMe(14:1(9Z)/22:1(13Z))	down	802.56	7.0373	2.14	0.93	0.0254
93	Cochliobolin A	down	859.53	7.0453	1.61	0.95	0.0442
94	LysoPI(16:0/0:0)	up	571.29	7.1068	1.47	1.04	0.0459
95	Hydroxy Ritonavir	up	757.28	7.0988	1.98	1.09	0.0292
96	PA(PGD2/8:0)	up	667.31	7.0988	1.03	1.02	0.0463
97	PE(16:0/18:0)	down	700.53	6.8628	1.91	0.94	0.0413
98	PE(16:1(9Z)/P-18:1(9Z))	down	698.51	6.5861	2.75	0.86	0.0448
99	PC(P-18:1(9Z)/18:0)	down	816.61	6.1907	2.05	0.94	0.0004

100	Palmitoylcarnitine	up	436.28	6.0412	1.38	1.03	0.0416
101	1,6-anhydro-N-acetyl-beta-muramate	up	295.07	5.7964	1.71	1.05	0.0032
102	Furanofukinin	up	293.18	5.7884	1.99	1.08	0.0026
103	Vitexin	up	431.10	4.5923	2.87	1.13	0.0005
104	N-Acetyl-DL-Methionine	up	190.05	3.0683	1.61	1.05	0.0195
105	Ethyl beta-D-fructofuranoside	up	461.19	2.4512	1.41	1.04	0.0330
106	Niflumic Acid	down	317.03	2.2672	1.09	0.98	0.0004
107	Oxypurinol	up	151.03	1.5053	1.85	1.06	0.0031
108	Orientin	up	447.09	4.3082	2.16	1.07	0.0010
109	PG(18:0/22:4(7Z,10Z,13Z,16Z))	down	861.55	6.1828	1.98	0.94	0.0052
110	3-Phosphoglycerate	down	184.99	0.5583	1.74	0.95	0.0056
111	Dodecanedioic Acid	down	229.14	5.7324	1.01	0.98	0.0339

Note: RT： retention time; m/z: mass to core ratio; VIP：The variable projection importance of the first principal component in the OPLS-DA model is an indicator for screening differential metabolites; FC(HS39/CON)：39 ℃ HS-CON group differential metabolite change multiple . P_value：The P-value of Student's t-test.

Table S3 List of differential metabolites in the 43 ℃ HS-43 ℃ DF group of MODE-K cells

Number	Metabolite name	Content changes	M/Z	RT	VIP	FC(HS39/CON)	P_value
1	Aspartame	up	295.13	2.8669	2.53	1.10	0.0161
2	DG(10:0/0:0/20:5(6E,8Z,11Z,14Z,17Z)-OH(5))	down	285.19	3.297	3.01	0.85	0.0016
3	Hispiduloside	up	463.12	4.2495	5.89	1.63	0.0000
4	Capsiamide	up	302.31	5.7482	1.15	1.02	0.0283
5	Prednisone acetate	up	418.22	5.7637	1.09	1.01	0.0364
6	PC(14:0/14:0)	up	678.51	5.865	2.44	1.08	0.0186
7	PC(14:0/0:0)	down	490.29	6.2632	2.89	0.88	0.0437
8	PE(16:1(9Z)/15:0)	up	708.51	6.3803	2.43	1.09	0.0401
9	S-Adenosylhomocysteine	up	385.13	1.6646	2.03	1.05	0.0111
10	Aglepristone	down	464.31	6.1072	1.52	0.97	0.0409
11	LysoPE(18:1(11Z)/0:0)	down	502.29	6.076	2.81	0.87	0.0446
12	PC(O-18:0/18:2(9Z,12Z))	up	772.62	6.0525	1.67	1.03	0.0385
13	Threonylserine	down	413.19	6.0057	1.52	0.96	0.0470
14	LysoPA(16:0/0:0)	down	452.28	5.9589	1.70	0.96	0.0249
15	Nuatigenin	down	494.32	5.8415	2.18	0.94	0.0058

16	Trans-Zeatin	up	261.14	2.5935	2.86	1.17	0.0197
17	Tuberculostearic acid	down	316.32	5.7637	3.62	0.89	0.0002
18	Isophorone	up	139.11	5.6078	1.00	1.01	0.0023
19	Chelidonine	up	336.12	4.3432	3.02	1.13	0.0000
20	Isovitexin	up	433.11	4.1559	3.92	1.19	0.0000
21	Luteolin-6-C-Glucoside	up	449.11	3.8279	3.63	1.16	0.0000
22	1-Hydroxy-6-methoxypyrene	up	266.12	4.8272	2.01	1.08	0.0103
23	3-Hydroxy-5,5,8a-Trimethyl-3,4,4a,6,7,8-Hexahydronaphthalene-2-Carboxylic Acid	up	261.14	2.7419	1.96	1.06	0.0374
24	1-Phenyl-1-cyclohexene	up	197.07	2.4539	3.13	1.17	0.0064
25	Gamma-Glutamyltyrosine	up	311.12	2.0389	2.57	1.11	0.0139
26	Pro-Pro-Pro	down	310.18	1.774	1.18	0.98	0.0199
27	Inosine 5'-Phosphate	up	349.05	1.5473	2.54	1.10	0.0066
28	Xanthosine 5'-Monophosphate	up	365.05	1.2114	4.72	1.42	0.0002
29	Imidazole Lactic Acid	up	157.06	0.6005	2.18	1.09	0.0116
30	Creatine	up	132.08	0.5926	1.64	1.03	0.0383
31	N-Acetyl-glucosamine 1-phosphate	up	324.05	0.5771	1.68	1.04	0.0482
32	Inosine 2'-phosphate	up	371.04	1.102	2.50	1.07	0.0027
33	PC(O-18:1(11Z)/16:0)	up	746.60	5.8806	1.09	1.01	0.0447
34	Dimethyl Sulfoxide	up	79.02	0.6629	6.40	1.72	0.0002
35	Gamma-Glutamylcysteine	up	251.07	1.0779	2.27	1.09	0.0209
36	Asp Ile Glu	down	376.17	2.0155	2.68	0.87	0.0415
37	Tuliposide A	down	296.14	2.3208	1.94	0.95	0.0029
38	Glutamylphenylalanine	up	293.11	3.4238	2.27	1.09	0.0213
39	Gabazine	up	332.13	3.8309	2.29	1.11	0.0440
40	Citicoline	up	510.09	4.3082	3.65	1.19	0.0000
41	Chrysophanein	up	461.11	4.6865	5.78	1.64	0.0000
42	Apigenin	up	269.05	5.6624	4.03	1.29	0.0001
43	PGH2	up	351.22	5.7164	1.41	1.03	0.0128
44	Trospectomycin	up	419.21	5.7164	1.61	1.04	0.0167
45	10-Hydroxydecanoic Acid	down	187.13	5.8044	1.43	0.97	0.0233
46	Pentadecanal	down	271.23	5.9782	1.49	0.96	0.0372
47	4-(undecan-5-yl)benzene-1-Sulfonic Acid	up	311.17	6.521	1.03	1.01	0.0148
48	Rocuronium	down	566.35	6.621	1.60	0.96	0.0461
49	Cochliobolin A	up	859.53	7.0453	2.17	1.07	0.0429
50	Dodecyl Hydrogen Sulfate	up	265.15	7.067	1.93	1.07	0.0160
51	PA(PGD2/8:0)	down	667.31	7.0988	2.04	0.94	0.0458
52	LysoPI(18:2(9Z,12Z)/0:0)	down	595.29	7.0988	3.25	0.84	0.0060

53	LysoPI(18:1(9Z)/0:0)	down	597.30	7.067	1.71	0.96	0.0204
54	PC(P-18:0/16:1(9Z))	up	788.58	6.7863	1.38	1.03	0.0156
55	Inosinic acid	up	347.04	1.0951	2.37	1.07	0.0065
56	1-(9Z-Nonadecenoyl)-glycero-3-phosphoethanolamine	down	538.31	6.0805	2.46	0.91	0.0042
57	XMP	up	363.04	1.1991	3.01	1.13	0.0095
58	PGA2	up	333.21	5.7164	1.47	1.03	0.0118
59	15-deoxy-delta-12,14-PGJ2	up	315.20	5.7164	1.59	1.04	0.0192
60	Histidylarginine	up	310.16	5.7004	1.00	1.02	0.0328
61	Suberic Acid	down	173.08	4.6603	1.38	0.97	0.0171
62	Vitexin	up	431.10	4.5923	3.48	1.15	0.0000
63	FAD	down	784.15	3.1929	1.50	0.97	0.0431
64	Ethyl beta-D-fructofuranoside	down	461.19	2.4512	1.54	0.96	0.0342
65	S-Adenosyl-L-homocysteine	up	383.11	1.8473	2.11	1.07	0.0062
66	Orientin	up	447.09	4.3082	3.52	1.15	0.0000
67	N-Acetyl-D-Glucosamine 6-Phosphate	up	300.05	0.5742	1.60	1.04	0.0311
68	Taurine	up	124.01	0.5504	3.18	1.19	0.0052
69	Glyceric Acid	down	105.02	0.5985	2.39	0.88	0.0402
70	9,10-Dihydroxystearic acid	down	297.24	5.9782	1.96	0.95	0.0088
71	Phenylacetylglutamine	up	309.11	2.5872	2.78	1.16	0.0176

Note: RT： retention time; m/z: mass to core ratio; VIP：The variable projection importance of the first principal component in the OPLS-DA model is an indicator for screening differential metabolites; FC(HS39/CON)：39 °C HS-CON group differential metabolite change multiple . P_value：The P-value of Student's t-test.

Table S4 Results of significant pathway enrichment analysis for the 39°C HS-39°C DF group

	Second Category	Pathway Desciption	Pvalue	Metabolite quantity	First Category
1	Organismal Systems	Sensory system	Taste transduction	0.0001	4
2	Human Diseases	Cancer: overview	Choline metabolism in cancer	0.0001	16
3	Metabolism	Lipid metabolism	Glycerophospholipid metabolism	0.0001	20
4	Metabolism	Carbohydrate metabolism	Amino sugar and nucleotide sugar metabolism	0.0003	7
5	Organismal Systems	Nervous system	Retrograde endocannabinoid signaling	0.0004	15

6	Environmental Information Processing	Signal transduction	FoxO signaling pathway	0.0006	2
7	Human Diseases	Infectious disease: parasitic	Leishmaniasis	0.0009	2
8	Metabolism	Glycan biosynthesis and metabolism	Glycosylphosphatidylinositol (GPI)-anchor biosynthesis	0.0009	4
9	Human Diseases	Substance dependence	Cocaine addiction	0.0013	2
10	Human Diseases	Substance dependence	Amphetamine addiction	0.0021	2
11	Metabolism	Energy metabolism	Sulfur metabolism	0.0021	3
12	Cellular Processes	Cell growth and death	Necroptosis	0.0027	2
13	Human Diseases	Substance dependence	Alcoholism	0.0027	2
14	Human Diseases	Cancer: overview	Central carbon metabolism in cancer	0.003	3
15	Human Diseases	Cardiovascular disease	Diabetic cardiomyopathy	0.0035	3
16	Metabolism	Global and overview maps	Biosynthesis of cofactors	0.0035	9
17	Metabolism	Global and overview maps	Biosynthesis of nucleotide sugars	0.0044	7
18	Human Diseases	Neurodegenerative disease	Amyotrophic lateral sclerosis	0.0053	2
19	Environmental Information Processing	Signal transduction	Sphingolipid signaling pathway	0.0061	2
20	Metabolism	Energy metabolism	Oxidative phosphorylation	0.0069	2

Table S5 Results of significant pathway enrichment analysis for the 41 °C HS-41 °C DF group

	First Category	Second Category	Pathway Desciption	Pvalue	Metabolite quantity
1	Metabolism	Lipid metabolism	Glycerophospholipid metabolism	0	10
2	Human Diseases	Cancer: overview	Choline metabolism in cancer	0.0022	4
3	Organismal Systems	Nervous system	Retrograde endocannabinoid signaling	0.0067	7
4	Metabolism	Lipid metabolism	Sphingolipid metabolism	0.0133	2
5	Metabolism	Lipid metabolism	Linoleic acid metabolism	0.0143	4
6	Human Diseases	Immune disease	Systemic lupus erythematosus	0.0197	1
7	Cellular Processes	Transport and catabolism	Autophagy - other	0.0197	4
8	Human Diseases	Cancer: overview	Central carbon metabolism in cancer	0.0242	2
9	Human Diseases	Infectious disease: viral	Kaposi sarcoma-associated herpesvirus infection	0.0325	4
10	Organismal Systems	Digestive system	Protein digestion and absorption	0.0378	2

11	Human Diseases	Infectious disease: parasitic	Leishmaniasis	0.0389	1
12	Cellular Processes	Transport and catabolism	Autophagy - animal	0.0389	4
13	Metabolism	Glycan biosynthesis and metabolism	Glycosylphosphatidylinositol (GPI)-anchor biosynthesis	0.0389	4
14	Metabolism	Amino acid metabolism	Glycine, serine and threonine metabolism	0.0393	2

Table S6 Results of significant pathway enrichment analysis for the 43℃ HS-43℃ DF group

	First Category	Second Category	Pathway Desciption	Pvalue	Metabolite quantity
1	Organismal Systems	Nervous system	Retrograde endocannabinoid signaling	0.0001	3
2	Metabolism	Lipid metabolism	Arachidonic acid metabolism	0.0004	4
3	Human Diseases	Cancer: overview	Choline metabolism in cancer	0.0012	2
4	Metabolism	Lipid metabolism	Glycerophospholipid metabolism	0.0022	3
5	Organismal Systems	Sensory system	Taste transduction	0.0099	3
6	Metabolism	Energy metabolism	Sulfur metabolism	0.0105	2
7	Cellular Processes	Transport and catabolism	Autophagy - other	0.0143	1
8	Organismal Systems	Nervous system	Serotonergic synapse	0.0167	2
9	Metabolism	Amino acid metabolism	Glycine, serine and threonine metabolism	0.0215	2
10	Human Diseases	Infectious disease: viral	Kaposi sarcoma-associated herpesvirus infection	0.0237	1
11	Cellular Processes	Transport and catabolism	Autophagy - animal	0.0283	1
12	Metabolism	Glycan biosynthesis and metabolism	Glycosylphosphatidylinositol (GPI)-anchor biosynthesis	0.0283	1
13	Metabolism	Global and overview maps	Nucleotide metabolism	0.0306	4

Table S7 List of key genes in the regulation of heat stress with mung bean polyphenols at 39°C

Gene_id	Gene name	FC(HS39/ CON)	Pvalue	Regulate	FC(DF39/ HS39)	Pvalue	Regulate
ENSMUSG00000024352	Spata24	3.6	9.37E-06	up	0.33	1.26E-05	down
ENSMUSG00000026271	Gpr35	9.07	2.67E-02	up	0.19	3.98E-02	down
ENSMUSG00000026809	Spaca9	2.69	2.30E-02	up	0.44	3.67E-02	down
ENSMUSG00000028463	Car9	9.14	1.83E-19	up	0.45	1.77E-05	down
ENSMUSG00000030680	Pagr1a	5.08	2.56E-02	up	0.32	2.44E-02	down
ENSMUSG00000031665	Sall1	29.06	3.67E-02	up	0.03	2.57E-02	down
ENSMUSG00000035237	Lcat	0.18	9.99E-04	down	0.03	4.40E-03	down
ENSMUSG00000036733	Rbm42	0.48	3.18E-05	down	4.35	5.27E-03	up
ENSMUSG00000042379	Esm1	3.88	3.34E-19	up	0.33	2.40E-17	down
ENSMUSG00000036502	Tmem255a	0.03	1.29E-02	down	25.00	3.24E-02	up
ENSMUSG00000048399	Tprg	5.79	4.85E-02	up	0.15	3.40E-02	down
ENSMUSG00000049313	Sorl1	0.04	3.68E-03	down	14.29	3.03E-02	up
ENSMUSG00000049233	Apoo-ps	0.11	7.66E-05	down	7.14	1.88E-03	up
ENSMUSG00000050982	Apol10a	0.05	4.11E-02	down	20.00	3.19E-02	up
ENSMUSG00000076609	Igkc	27.25	4.39E-02	up	0.03	3.11E-02	down
ENSMUSG00000090877	Hspa1b	0.13	1.70E-02	down	16.67	7.45E-05	up
ENSMUSG00000058385	H2bc8	0.35	2.48E-02	down	7.14	3.55E-08	up
ENSMUSG00000069268	H2bc7	0.28	3.81E-07	down	5.88	1.50E-12	up
ENSMUSG00000093769	H3c14	0.01	2.94E-05	down	33.33	5.67E-03	up
ENSMUSG00000079262	Slco1a6	5.99	9.95E-04	up	0.20	3.28E-03	down
ENSMUSG00000091498	Mpc1-ps	2.24	6.16E-03	up	0.09	1.17E-09	down
ENSMUSG00000096438	Gapdh-ps15	4.18	3.41E-02	up	0.25	3.35E-02	down
ENSMUSG00000097530	Kansl2-ps	3.44	6.38E-03	up	0.09	3.96E-02	down
ENSMUSG00000098404	Mrip-ps	3.54	3.29E-03	up	0.28	6.20E-04	down
ENSMUSG00000116165	Pdxp	0.01	1.23E-06	down	0.02	1.14E-09	up
ENSMUSG00000053117	E330013P04Rik	3.5	1.29E-02	up	0.30	1.13E-02	down
ENSMUSG00000108912	E230020D15Rik	3.3	1.34E-02	up	0.44	4.57E-02	down
ENSMUSG00000112489	9230116L04Rik	0.08	1.84E-02	down	14.29	4.82E-03	up
ENSMUSG00000084792	1700056N10Rik	0.19	1.97E-02	down	5.88	2.38E-03	up
ENSMUSG00000050299	Gm9843	3.6	1.38E-02	up	0.46	4.02E-02	down
ENSMUSG00000066647	Gm5113	0.43	3.28E-02	down	2.44	1.51E-02	up
ENSMUSG00000068165	Gm10233	40.49	3.17E-02	up	0.02	2.46E-02	down
ENSMUSG00000083367	Gm8806	0.22	4.90E-02	down	5.26	1.39E-02	up
ENSMUSG00000084858	Gm1980	2.38	4.53E-02	up	0.43	1.38E-02	down

ENSMUSG00000086364	Gm11751	30.12	1.40E-03	up	0.25	4.75E-02	down
ENSMUSG00000091509	Gm17066	0.39	2.82E-03	down	2.13	2.34E-02	up
ENSMUSG00000095847	Gm5451	3.23	1.78E-03	up	0.09	2.44E-02	down
ENSMUSG00000099190	Gm27188	4.97	4.03E-04	up	0.09	1.42E-07	down
ENSMUSG00000105102	Gm35507	4.11	2.44E-02	up	0.31	4.22E-02	down
ENSMUSG00000106568	Gm42814	0.2	4.04E-03	down	3.23	4.81E-02	up
ENSMUSG00000107603	Gm43921	0.19	4.09E-02	down	5.00	4.09E-02	up
ENSMUSG00000107928	Gm45140	0.41	3.89E-02	down	2.44	5.85E-07	up
ENSMUSG00000108436	Gm44851	2.87	3.13E-02	up	0.39	4.32E-02	down
ENSMUSG00000110588	Gm45774	0.44	6.20E-03	down	2.94	5.97E-04	up
ENSMUSG00000113555	Gm10095	2.95	2.96E-02	up	0.23	3.12E-03	down
ENSMUSG00000114378	Gm49355	0.35	4.56E-02	down	3.33	1.38E-02	up
ENSMUSG00000114898	Gm49390	10.01	3.12E-02	up	0.12	3.42E-02	down
ENSMUSG00000117477	Gm50092	29.35	1.01E-02	up	0.06	1.94E-02	down
ENSMUSG00000072769	Gm10419	2.34	1.58E-03	up	0.47	4.57E-03	down
ENSMUSG00000072930	Gm15107	0.45	1.92E-02	down	2.38	3.24E-03	up

Table S8 List of key genes in the regulation of heat stress with mung bean polyphenols at 41°C

Gene_id	Gene name	FC(HS41/ CON)	Pvalue	Regulate	FC(DF41/ HS41)	Pvalue	Regulate
ENSMUSG00000000204	Slfn4	15.49	6.04E-07	up	0.46	2.15E-02	down
ENSMUSG00000001029	Icam2	0.04	2.25E-03	down	16.67	1.91E-02	up
ENSMUSG00000021750	Fam107a	0.15	7.00E-20	down	0.41	2.51E-02	down
ENSMUSG00000022586	Ly6i	79.71	1.19E-05	up	0.26	1.36E-02	down
ENSMUSG00000024172	St6gal2	0.24	1.95E-02	down	4.55	1.13E-02	up
ENSMUSG00000024770	Lipn	0.24	2.53E-03	down	2.86	4.12E-02	up
ENSMUSG00000028524	Sgip1	2.3	5.93E-03	up	0.26	1.58E-04	down
ENSMUSG00000029049	Morn1	0.47	1.83E-02	down	2.50	2.74E-03	up
ENSMUSG00000029352	Crybb3	0.05	9.13E-03	down	16.63	1.80E-02	up
ENSMUSG00000036305	Rpl39-ps	0.32	1.13E-03	down	7.69	1.01E-04	up
ENSMUSG00000040280	Ndufa4l2	0.12	9.40E-03	down	6.25	3.58E-02	up
ENSMUSG00000043633	Fam221b	0.14	6.45E-03	down	5.26	2.25E-02	up
ENSMUSG00000043953	Ccrl2	12.77	1.11E-06	up	0.41	1.11E-02	down
ENSMUSG00000044548	Dact1	0.09	2.99E-03	down	6.67	2.47E-02	up
ENSMUSG00000052271	Bhlha15	0.04	3.83E-03	down	16.66	1.50E-02	up
ENSMUSG00000060183	Cxcl11	147.51	5.37E-08	up	0.43	2.11E-02	down
ENSMUSG00000060397	Zfp128	0.43	6.34E-04	down	2.22	1.11E-03	up
ENSMUSG00000062456	Rpl9-ps6	0.19	2.78E-33	down	3.57	4.05E-11	up
ENSMUSG00000063556	Gm10132	0.38	1.21E-06	down	3.45	3.46E-03	up

ENSMUSG00000066632	Pgk1-rs7	4.22	9.24E-09	up	0.40	4.86E-02	down
ENSMUSG00000069188	Gm13192	0.05	2.09E-02	down	14.29	4.54E-02	up
ENSMUSG00000071532	Gm10335	0.34	1.99E-07	down	3.13	3.93E-08	up
ENSMUSG00000072693	Gm10401	0.15	3.28E-02	down	2.86	4.51E-02	up
ENSMUSG00000073600	Prob1	0.14	1.29E-02	down	7.14	2.59E-08	up
ENSMUSG00000074673	Ttll9	0.14	3.75E-02	down	6.25	3.91E-02	up
ENSMUSG00000077306	Gm22469	0.12	5.86E-03	down	5.88	4.03E-02	up
ENSMUSG00000082035	Rpl17-ps8	0.3	9.39E-12	down	3.03	1.11E-07	up
ENSMUSG00000083152	Apc-ps1	0.03	1.54E-03	down	14.29	3.13E-02	up
ENSMUSG00000087624	9230111E07Rik	0.04	1.69E-02	down	50.00	6.08E-04	up
ENSMUSG00000092036	Gm2244	0.02	1.18E-05	down	10.00	4.71E-02	up
ENSMUSG00000092072	Gm4540	0.03	2.84E-03	down	20.00	6.52E-07	up
ENSMUSG00000095887	Gm10096	0.29	1.67E-05	down	3.33	1.82E-05	up
ENSMUSG00000101122	Gm17971	0.11	4.16E-03	down	7.14	1.37E-02	up
ENSMUSG00000103558	Gm38220	3.05	4.90E-02	up	0.36	4.70E-02	down
ENSMUSG00000103887	Gm37008	0.15	1.71E-02	down	5.26	4.68E-02	up
ENSMUSG00000105339	Gm42457	0.03	2.50E-03	down	25.00	1.84E-02	up
ENSMUSG00000105796	Gm42845	22.13	2.42E-02	up	0.04	1.73E-02	down
ENSMUSG00000106574	Gm2451	4.5	3.60E-04	up	0.43	1.06E-02	down
ENSMUSG00000110679	Rpl10-ps5	0.02	7.70E-04	down	25.00	2.91E-02	up
ENSMUSG00000112392	Gm35240	0.43	2.06E-02	down	2.78	8.41E-03	up
ENSMUSG00000114905	Gm48113	47.55	4.96E-04	up	2.38	4.21E-02	up
ENSMUSG00000115129	Gm48916	0.24	7.78E-03	down	3.13	3.09E-02	up
ENSMUSG00000116835	Gm49594	0.35	5.53E-41	down	2.38	5.28E-16	up
ENSMUSG00000116908	Gm49599	0.09	9.01E-20	down	2.22	3.66E-02	up
ENSMUSG00000117905	Gm50230	0.12	1.17E-02	down	9.09	7.74E-03	up
ENSMUSG00002076173	Gm55118	2.57	2.75E-02	up	2.13	1.13E-02	up
ENSMUSG00000025408	Ddit3	0.45	2.32E-40	down	3.57	1.87E-04	up

Table S9 List of key genes in the regulation of heat stress with mung bean polyphenols at 43°C

Gene_id	Gene name	FC(HS43/ CON)	Pvalue	Regulate	FC(DF43/ HS43)	Pvalue	Regulate
ENSMUSG00000000386	Mx1	3.83	7.40E-04	up	0.46	3.23E-02	down
ENSMUSG00000003379	Cd79a	29.70	1.56E-09	up	0.47	3.38E-02	down
ENSMUSG00000003477	Inmt	0.15	3.00E-16	down	0.40	3.88E-02	down
ENSMUSG00000006310	Zbtb32	3.44	6.43E-03	up	0.41	3.83E-02	down
ENSMUSG00000006311	Etv2	65.86	3.11E-04	up	0.02	5.12E-04	down
ENSMUSG00000006378	Gcat	2.45	1.77E-17	up	0.41	1.77E-17	down
ENSMUSG00000006411	Nectin4	20.86	3.63E-03	up	0.23	4.16E-02	down
ENSMUSG00000006469	Slc34a3	43.66	9.17E-03	up	0.05	2.57E-02	down
ENSMUSG00000007030	Vwa7	12.37	9.18E-04	up	0.26	2.63E-02	down
ENSMUSG00000009350	Mpo	11.04	4.70E-03	up	0.10	5.06E-03	down
ENSMUSG00000012042	4930579F01Rik	34.69	3.05E-03	up	0.12	1.93E-02	down
ENSMUSG00000014773	Dll1	33.68	1.16E-06	up	0.33	2.44E-02	down
ENSMUSG00000017344	Vtn	109.53	9.85E-06	up	0.25	2.67E-02	down
ENSMUSG00000017737	Mmp9	19.64	8.42E-04	up	0.17	1.15E-02	down
ENSMUSG00000018341	Il12rb2	65.13	4.57E-06	up	0.32	3.19E-02	down
ENSMUSG00000019647	Sema6a	324.02	1.75E-09	up	0.24	2.90E-03	down
ENSMUSG00000020383	Il13	30.02	1.85E-02	up	0.07	4.56E-02	down
ENSMUSG00000020609	Apob	111.25	2.26E-05	up	0.22	5.00E-02	down
ENSMUSG00000020627	Klhl29	6.81	4.30E-02	up	0.10	1.42E-02	down
ENSMUSG00000020912	Krt12	64.12	1.56E-11	up	0.48	2.07E-02	down
ENSMUSG00000021135	Slc10a1	47.34	1.15E-05	up	0.19	1.80E-03	down
ENSMUSG00000021298	Gpr132	17.89	8.44E-03	up	0.12	2.07E-02	down
ENSMUSG00000021314	Amph	24.02	4.04E-02	up	3.53	4.16E-02	up
ENSMUSG00000021363	Mak	5.09	7.23E-03	up	2.33	2.06E-02	up
ENSMUSG00000021590	Spata9	3.64	3.62E-04	up	0.45	9.34E-03	down
ENSMUSG00000021872	Rnase10	117.44	7.65E-06	up	0.22	2.40E-02	down
ENSMUSG00000022454	Nell2	981.22	9.55E-12	up	0.37	2.86E-02	down
ENSMUSG00000022619	Mapk8ip2	6.55	4.29E-03	up	0.26	1.54E-02	down
ENSMUSG00000022803	Popdc2	9.94	4.73E-05	up	0.32	2.80E-02	down
ENSMUSG00000022853	Ehhadh	82.58	3.14E-05	up	0.10	1.08E-03	down
ENSMUSG00000022878	Adipoq	31.65	3.84E-03	up	0.13	3.01E-02	down
ENSMUSG00000023903	Mmp25	35.10	1.19E-05	up	0.29	2.89E-02	down
ENSMUSG00000023914	Mep1a	4.72	6.29E-05	up	0.42	1.80E-02	down
ENSMUSG00000024114	Prss41	31.96	1.50E-02	up	0.03	1.42E-02	down
ENSMUSG00000024842	Cabp4	17.52	3.94E-14	up	0.27	3.96E-02	down
ENSMUSG00000025014	Dntt	149.37	7.50E-07	up	0.25	1.57E-02	down
ENSMUSG00000025141	Myadml2	31.13	8.27E-04	up	0.05	1.26E-03	down

ENSMUSG00000025348	Itga7	6.68	1.58E-03	up	0.28	4.30E-02	down
ENSMUSG00000025408	Ddit3	0.34	7.21E-05	down	2.94	7.21E-05	up
ENSMUSG00000025977	Boll	3.65	3.84E-03	up	0.32	1.30E-02	down
ENSMUSG00000026173	Plcd4	4.28	4.91E-07	up	0.39	5.22E-04	down
ENSMUSG00000026175	Vil1	12.83	2.21E-04	up	0.24	2.05E-02	down
ENSMUSG00000026582	Sele	0.46	2.91E-03	down	0.49	5.60E-03	down
ENSMUSG00000026725	Tnn	6.05	1.02E-05	up	0.33	1.43E-02	down
ENSMUSG00000027360	Hdc	22.68	7.82E-06	up	0.31	1.62E-02	down
ENSMUSG00000027880	Slc25a54	7.79	6.16E-03	up	0.04	7.79E-04	down
ENSMUSG00000027967	Neurog2	896.85	6.52E-09	up	0.25	2.69E-02	down
ENSMUSG00000028012	Rrh	2.75	3.74E-04	up	0.43	2.10E-03	down
ENSMUSG00000028860	Sytl1	6.52	5.29E-05	up	0.46	2.77E-02	down
ENSMUSG00000029193	Cckar	204.96	5.54E-08	up	0.24	8.09E-03	down
ENSMUSG00000029368	Alb	49.64	4.87E-03	up	0.08	3.39E-02	down
ENSMUSG00000029641	Rasl11a	84.62	3.80E-07	up	0.45	1.00E-03	down
ENSMUSG00000029648	Flt1	5.46	5.26E-15	up	0.49	3.25E-02	down
ENSMUSG00000030087	Klf15	0.23	6.88E-03	down	0.05	2.19E-02	down
ENSMUSG00000030214	Plbd1	137.51	1.30E-06	up	0.25	2.97E-02	down
ENSMUSG00000030278	Cidec	24.97	8.65E-07	up	0.32	2.84E-02	down
ENSMUSG00000030329	Pianp	4.91	1.24E-04	up	0.23	4.01E-04	down
ENSMUSG00000030468	Siglecg	0.37	2.04E-02	down	0.20	2.47E-02	down
ENSMUSG00000030653	Gm45837	69.77	2.34E-04	up	0.08	9.83E-03	down
ENSMUSG00000030786	Itgam	99.46	2.82E-05	up	0.04	1.11E-03	down
ENSMUSG00000030895	Hpx	3.28	2.84E-02	up	0.18	2.63E-03	down
ENSMUSG00000031227	Magee1	2.63	5.71E-03	up	0.26	4.30E-04	down
ENSMUSG00000031326	Cdx4	57.73	8.57E-04	up	0.07	1.12E-02	down
ENSMUSG00000031637	Lrp2bp	13.84	9.98E-09	up	0.47	3.06E-02	down
ENSMUSG00000031932	Gpr83	179.21	2.10E-07	up	0.16	1.35E-03	down
ENSMUSG00000032238	Rora	6.33	7.28E-04	up	3.66	1.89E-08	up
ENSMUSG00000032297	Celf6	42.11	4.82E-04	up	0.12	7.48E-03	down
ENSMUSG00000032517	Mobp	47.93	6.97E-04	up	0.21	4.06E-02	down
ENSMUSG00000032719	Sbspon	3.80	4.33E-04	up	0.39	1.20E-02	down
ENSMUSG00000032911	Cspg4	2.33	1.66E-02	up	0.34	1.96E-02	down
ENSMUSG00000033187	BC016579	95.28	1.40E-04	up	0.08	8.26E-03	down
ENSMUSG00000033368	Trim69	38.40	2.14E-18	up	0.47	8.42E-03	down
ENSMUSG00000033644	Piwil2	5.04	1.32E-02	up	0.11	1.41E-03	down
ENSMUSG00000034683	Ppp1r1c	115.15	2.98E-05	up	0.22	4.47E-02	down
ENSMUSG00000034764	1700006J14Rik	0.02	3.12E-04	down	30.45	1.17E-02	up
ENSMUSG00000035576	L3mbtl1	143.25	1.61E-06	up	0.22	1.27E-02	down
ENSMUSG00000036305	Rpl39-ps	2.93	4.85E-04	up	2.10	2.05E-05	up
ENSMUSG00000036452	Arhgap26	0.48	8.29E-12	down	2.09	8.29E-12	up
ENSMUSG00000036655	Colec11	19.00	8.92E-04	up	0.24	2.20E-02	down
ENSMUSG00000037346	Hrh4	69.87	2.01E-04	up	0.06	3.93E-03	down
ENSMUSG00000037548	H2-DMb2	115.23	2.10E-05	up	0.14	7.51E-03	down

ENSMUSG00000037727	Avp	39.25	3.87E-03	up	0.36	1.70E-02	down
ENSMUSG00000038199	Iqca1l	49.63	5.03E-03	up	0.04	1.63E-02	down
ENSMUSG00000038305	Spats2l	2.18	6.71E-03	up	0.49	7.91E-03	down
ENSMUSG00000038390	Gpr162	0.38	2.68E-02	down	2.73	6.71E-03	up
ENSMUSG00000038932	Tcfl5	4.40	3.34E-02	up	0.09	4.87E-03	down
ENSMUSG00000038980	Rbbp8nl	25.86	3.76E-02	up	0.04	3.61E-02	down
ENSMUSG00000039239	Tgfb2	13.60	1.81E-03	up	0.20	2.96E-02	down
ENSMUSG00000039579	Grin3a	3.47	2.50E-02	up	0.36	4.79E-02	down
ENSMUSG00000039981	Zc3h12d	35.42	2.61E-05	up	0.18	1.11E-02	down
ENSMUSG00000040017	Saa4	87.34	2.18E-04	up	0.09	9.09E-03	down
ENSMUSG00000040046	Tph1	3.59	3.48E-02	up	0.20	1.34E-02	down
ENSMUSG00000040247	Tbc1d10c	3.13	8.94E-03	up	0.36	2.16E-02	down
ENSMUSG00000040283	Btnl9	35.05	6.36E-05	up	0.04	2.21E-04	down
ENSMUSG00000040367	Lrrd1	14.43	5.85E-05	up	0.20	1.18E-02	down
ENSMUSG00000041073	Nacad	16.13	5.54E-08	up	0.27	8.63E-04	down
ENSMUSG00000041117	Ccdc8	19.14	1.59E-05	up	0.25	4.30E-03	down
ENSMUSG00000041872	Il17f	73.86	1.90E-08	up	0.43	1.82E-03	down
ENSMUSG00000042250	Pglyrp4	26.26	7.77E-04	up	0.05	1.18E-03	down
ENSMUSG00000042678	Myo15	3.19	9.59E-03	up	0.39	2.21E-02	down
ENSMUSG00000042707	Dnali1	24.34	3.35E-07	up	0.49	4.79E-02	down
ENSMUSG00000042988	Notum	43.67	8.88E-03	up	0.02	9.00E-03	down
ENSMUSG00000043410	Hfm1	95.21	6.10E-04	up	0.14	1.28E-02	down
ENSMUSG00000043441	Gpr149	0.13	4.84E-02	down	8.07	1.80E-02	up
ENSMUSG00000043456	Zfp536	231.59	4.43E-09	up	0.48	4.75E-02	down
ENSMUSG00000043670	Diras1	59.29	5.58E-08	up	0.37	3.15E-02	down
ENSMUSG00000043925	Olfr544	9.54	1.83E-02	up	0.08	1.11E-02	down
ENSMUSG00000044176	Spink10	4.02	7.26E-03	up	0.31	1.78E-02	down
ENSMUSG00000044724	Gpr152	42.91	1.32E-06	up	0.35	4.40E-02	down
ENSMUSG00000045034	Ankrd34b	148.96	3.74E-06	up	0.12	6.31E-03	down
ENSMUSG00000045350	Fam186a	15.36	3.69E-04	up	0.29	3.88E-02	down
ENSMUSG00000045551	Fpr1	3.41	1.95E-03	up	0.49	3.81E-02	down
ENSMUSG00000046213	Cym	20.42	2.70E-02	up	0.05	2.73E-02	down
ENSMUSG00000046487	Mospd4	12.58	3.08E-03	up	0.16	1.50E-02	down
ENSMUSG00000047021	Cfap65	45.68	5.63E-03	up	0.05	1.81E-02	down
ENSMUSG00000047384	A730013G03Rik	69.66	2.80E-04	up	0.11	1.15E-02	down
ENSMUSG00000047419	Cmya5	4.89	1.48E-02	up	0.32	4.64E-02	down
ENSMUSG00000047420	Fam180a	5.19	2.33E-04	up	0.39	1.76E-02	down
ENSMUSG00000047953	Gp5	9.21	1.88E-03	up	0.27	2.38E-02	down
ENSMUSG00000048644	Ctxn1	3.48	3.12E-03	up	0.46	4.74E-02	down
ENSMUSG00000048772	Tmem53	0.26	2.27E-02	down	0.04	1.93E-02	down
ENSMUSG00000048960	Prex2	5.26	1.08E-03	up	0.43	4.51E-02	down
ENSMUSG00000050217	Lgsn	113.41	9.34E-06	up	0.26	2.78E-02	down
ENSMUSG00000050359	Sprr1a	51.85	1.62E-03	up	0.12	3.08E-02	down
ENSMUSG00000050578	Mmp13	0.33	1.02E-06	down	0.39	1.59E-03	down

ENSMUSG00000050612	Txndc2	7.20	2.66E-03	up	0.31	4.66E-02	down
ENSMUSG00000051243	Islr2	15.99	2.30E-06	up	0.25	1.17E-02	down
ENSMUSG00000051498	Tlr6	0.40	1.34E-06	down	0.41	1.54E-03	down
ENSMUSG00000051639	Fbl-ps2	3.00	2.90E-02	up	2.38	1.73E-02	up
ENSMUSG00000051648	Kctd19	722.67	1.51E-09	up	0.41	7.45E-03	down
ENSMUSG00000052013	Btla	107.33	2.20E-05	up	0.07	1.53E-03	down
ENSMUSG00000052160	Pld4	7.81	1.44E-03	up	0.21	2.67E-02	down
ENSMUSG00000052270	Fpr2	152.51	4.19E-08	up	0.38	7.51E-03	down
ENSMUSG00000053687	Dpep2	0.29	8.14E-11	down	3.46	8.14E-11	up
ENSMUSG00000053863	Mepe	39.91	5.31E-03	up	0.10	4.65E-02	down
ENSMUSG00000054252	Fgfr3	21.22	6.19E-03	up	0.16	3.02E-02	down
ENSMUSG00000054320	Lrrc36	305.74	1.37E-10	up	0.44	1.18E-02	down
ENSMUSG00000054360	Bsx	119.33	8.16E-06	up	0.22	3.79E-02	down
ENSMUSG00000054555	Adam12	6.57	4.14E-04	up	0.33	4.03E-02	down
ENSMUSG00000054679	Srsf12	25.87	3.58E-02	up	0.04	3.52E-02	down
ENSMUSG00000054892	Txk	46.01	2.33E-03	up	0.09	2.28E-02	down
ENSMUSG00000055102	Zfp819	16.30	2.27E-02	up	0.10	4.06E-02	down
ENSMUSG00000055216	9430025C20Rik	27.39	4.12E-10	up	0.32	2.26E-03	down
ENSMUSG00000056900	Usp13	77.54	3.15E-05	up	0.12	1.55E-03	down
ENSMUSG00000058254	Tspan7	15.39	4.92E-02	up	0.07	4.90E-02	down
ENSMUSG00000058743	Kcnj14	75.37	1.45E-10	up	0.44	4.10E-03	down
ENSMUSG00000058809	Hspd1-ps3	45.76	3.87E-32	up	0.48	3.55E-04	down
ENSMUSG00000059213	Ddn	15.94	1.08E-10	up	0.49	3.87E-02	down
ENSMUSG00000059562	Ccdc154	16.19	1.87E-04	up	0.31	3.94E-02	down
ENSMUSG00000064225	Paqr9	137.13	4.25E-06	up	0.13	5.35E-03	down
ENSMUSG00000064288	H4c12	36.16	8.71E-05	up	0.35	3.52E-02	down
ENSMUSG00000064360	mt-Nd3	0.46	1.26E-09	down	0.33	2.67E-03	down
ENSMUSG00000065701	Rny1	9.01	4.44E-03	up	2.19	4.80E-02	up
ENSMUSG00000065947	mt-Nd4l	0.20	2.91E-08	down	3.77	2.27E-04	up
ENSMUSG00000067338	Tuba3b	417.82	7.00E-12	up	0.37	4.78E-04	down
ENSMUSG00000067795	4930444P10Rik	61.37	3.07E-04	up	0.43	2.16E-02	down
ENSMUSG00000068349	Gml	161.23	4.64E-07	up	0.30	4.57E-02	down
ENSMUSG00000068740	Celsr2	33.05	9.20E-07	up	0.35	3.64E-02	down
ENSMUSG00000069816	Olfr23	29.76	4.15E-02	up	0.04	4.10E-02	down
ENSMUSG00000070330	Tmem235	27.57	7.34E-03	up	0.11	4.32E-02	down
ENSMUSG00000070720	Tmem200b	41.71	1.01E-02	up	0.05	2.82E-02	down
ENSMUSG00000071226	Cecr2	20.85	3.14E-03	up	0.39	1.27E-02	down
ENSMUSG00000073408	Mucl3	27.79	5.47E-03	up	0.15	4.18E-02	down
ENSMUSG00000073414	Mpig6b	147.56	4.45E-08	up	0.49	3.87E-02	down
ENSMUSG00000073600	Prob1	0.06	3.99E-07	down	5.56	2.27E-02	up
ENSMUSG00000073739	Gm16287	4.76	1.36E-02	up	0.22	1.39E-02	down
ENSMUSG00000073761	4933427I04Rik	8.63	4.99E-04	up	0.24	6.81E-03	down
ENSMUSG00000074388	Gm5544	41.81	1.42E-03	up	0.10	1.28E-02	down
ENSMUSG00000074483	Bglap	42.19	3.92E-03	up	0.09	3.26E-02	down

ENSMUSG00000074657	Kif5a	9.20	4.19E-06	up	0.37	4.98E-02	down
ENSMUSG00000078153	Psme2b	34.35	2.18E-02	up	0.03	2.39E-02	down
ENSMUSG00000078606	Gvin2	0.09	1.65E-13	down	0.42	5.09E-04	down
ENSMUSG00000079484	Phyhd1	0.10	1.79E-43	down	0.44	1.51E-02	down
ENSMUSG00000080021	Gm5915	49.01	3.79E-04	up	0.18	1.85E-02	down
ENSMUSG00000081272	Ap2m1-ps	5.45	1.70E-04	up	0.35	2.25E-02	down
ENSMUSG00000081605	Gm15953	14.62	7.70E-07	up	0.38	1.96E-02	down
ENSMUSG00000081965	Gm11620	3.53	3.27E-03	up	0.28	2.22E-03	down
ENSMUSG00000082741	Gm9703	32.48	1.79E-04	up	0.29	2.73E-02	down
ENSMUSG00000083899	Gm12346	2.37	3.52E-02	up	0.35	1.34E-02	down
ENSMUSG00000086179	Gm14317	4.62	2.23E-07	up	0.46	5.29E-03	down
ENSMUSG00000086187	Gm12860	96.40	1.73E-05	up	2.16	4.50E-02	up
ENSMUSG00000086231	Rapgef4os3	41.65	2.30E-02	up	0.03	2.39E-02	down
ENSMUSG00000086275	1700121C08Rik	8.85	1.37E-05	up	0.29	1.85E-02	down
ENSMUSG00000086413	Gm12415	21.61	1.93E-06	up	0.40	2.03E-02	down
ENSMUSG00000087141	Plcxd2	4.65	2.04E-03	up	0.34	3.86E-02	down
ENSMUSG00000087223	4930442L01Rik	51.79	1.95E-03	up	0.11	3.37E-02	down
ENSMUSG00000087231	E230016M11Rik	0.35	3.97E-06	down	0.45	8.42E-03	down
ENSMUSG00000087445	Gm14286	0.09	1.06E-10	down	0.38	1.60E-02	down
ENSMUSG00000088088	Rmrp	277.25	1.04E-159	up	0.00	1.04E-159	down
ENSMUSG00000089669	Tnfsf13	0.42	3.14E-02	down	0.24	4.02E-02	down
ENSMUSG00000089670	Gm16581	211.49	8.00E-09	up	0.41	3.42E-02	down
ENSMUSG00000089697	Gm15947	42.03	4.09E-03	up	0.05	1.43E-02	down
ENSMUSG00000089961	Gm16567	0.20	1.78E-05	down	0.27	4.42E-02	down
ENSMUSG00000091709	Gm17189	37.92	6.67E-03	up	0.06	2.08E-02	down
ENSMUSG00000091983	Olfr457	11.97	7.97E-03	up	0.09	7.04E-03	down
ENSMUSG00000092253	H2-Q3	77.45	3.26E-04	up	0.15	3.70E-02	down
ENSMUSG00000092518	Garin5b	434.13	2.55E-11	up	0.39	1.41E-02	down
ENSMUSG00000093402	Gm18588	0.02	8.11E-05	down	33.61	4.61E-03	up
ENSMUSG00000093445	Lrch4	0.11	2.66E-36	down	5.04	2.65E-15	up
ENSMUSG00000094840	Muc3a	248.63	1.18E-08	up	0.31	1.58E-02	down
ENSMUSG00000095621	Gm15085	0.00	3.24E-10	down	81.79	2.49E-05	up
ENSMUSG00000097233	Gm17552	420.46	3.10E-11	up	0.33	2.96E-03	down
ENSMUSG00000097333	Zfp87	0.50	1.87E-06	down	0.48	1.70E-02	down
ENSMUSG00000097471	5830432E09Rik	4.37	9.31E-03	up	0.21	7.05E-03	down
ENSMUSG00000097619	4833422M21Rik	10.13	2.12E-04	up	0.36	4.21E-02	down
ENSMUSG00000097715	Gpr137b-ps	0.19	1.09E-05	down	2.75	2.09E-02	up
ENSMUSG00000099032	Tcf24	8.54	5.69E-04	up	0.28	2.79E-02	down
ENSMUSG00000099102	Gm11983	145.54	7.46E-07	up	0.33	2.80E-02	down
ENSMUSG00000101791	2210011K15Rik	2.48	1.72E-02	up	0.32	3.96E-03	down
ENSMUSG00000102828	Gm38182	0.45	9.10E-03	down	2.46	2.16E-03	up
ENSMUSG00000102908	Gm7558	24.01	4.56E-02	up	0.05	4.41E-02	down
ENSMUSG00000105851	9130604C24Rik	8.69	4.44E-04	up	0.22	7.83E-03	down
ENSMUSG00000106188	Gm9710	0.28	1.40E-03	down	0.34	8.77E-04	down

ENSMUSG00000108030	9530062K07Rik	9.59	6.30E-06	up	0.30	3.75E-02	down
ENSMUSG00000108079	Gm44210	8.87	7.31E-06	up	0.25	1.35E-03	down
ENSMUSG00000108236	0610033M10Rik	264.04	1.89E-04	up	0.19	6.45E-03	down
ENSMUSG00000108368	Gm45053	3.79	1.10E-02	up	0.34	4.92E-02	down
ENSMUSG00000108774	Gm45136	43.74	1.97E-02	up	0.03	2.36E-02	down
ENSMUSG00000109032	Gm7972	39.70	1.27E-02	up	0.03	1.28E-02	down
ENSMUSG00000109299	Gm45164	23.87	4.94E-02	up	0.05	4.74E-02	down
ENSMUSG00000110080	Gm6145	9.41	1.36E-05	up	0.24	4.12E-03	down
ENSMUSG00000110104	Gm45717	9.89	1.16E-03	up	0.20	2.42E-02	down
ENSMUSG00000110368	Gm45518	3.59	1.70E-03	up	0.46	3.13E-02	down
ENSMUSG00000110492	Gm5358	39.93	5.30E-03	up	0.10	4.09E-02	down
ENSMUSG00000110619	Gm7850	164.74	2.52E-07	up	0.38	4.14E-02	down
ENSMUSG00000110679	Rpl10-ps5	3.38	1.63E-02	up	0.28	3.36E-02	down
ENSMUSG00000111028	Gm5922	33.10	5.24E-07	up	0.38	2.23E-02	down
ENSMUSG00000111137	Gm2553	35.77	1.29E-02	up	0.06	3.43E-02	down
ENSMUSG00000111683	Gm49367	56.07	9.14E-04	up	2.70	4.41E-02	up
ENSMUSG00000112719	Gm45925	9.17	9.46E-03	up	0.16	3.52E-02	down
ENSMUSG00000114196	Gm47547	0.43	1.08E-03	down	0.44	3.11E-02	down
ENSMUSG00000114231	Gm40968	3.00	3.87E-02	up	0.27	1.42E-02	down
ENSMUSG00000114245	Percc1	57.74	8.42E-04	up	0.17	4.94E-02	down
ENSMUSG00000115852	Gm52969	0.04	4.59E-02	down	21.52	4.59E-02	up
ENSMUSG00000116534	Gm49731	21.43	2.01E-02	up	0.09	4.50E-02	down
ENSMUSG00000116831	Gm30505	35.97	9.94E-03	up	0.06	2.83E-02	down
ENSMUSG00000116994	Gm49684	15.42	4.45E-02	up	0.04	1.71E-02	down
ENSMUSG00000117257	Gm4948	38.02	6.37E-03	up	3.16	2.66E-02	up
ENSMUSG00000117312	Gm49931	21.29	1.04E-04	up	0.16	8.10E-03	down
ENSMUSG00000117525	Gm50034	135.70	1.51E-06	up	0.31	3.63E-02	down
ENSMUSG00000117780	Gm3734	6.91	1.44E-05	up	0.17	1.85E-04	down
ENSMUSG00000117922	Gm50397	37.84	7.73E-03	up	0.03	7.66E-03	down
ENSMUSG00000117923	Gm30593	297.98	3.70E-05	up	0.08	1.34E-02	down
ENSMUSG00000117966	1700061A03Rik	50.12	2.30E-04	up	0.22	2.50E-02	down
ENSMUSG00000117988	Gm8663	62.25	4.40E-04	up	0.13	2.98E-02	down
ENSMUSG00000118030	Gm50163	8.92	1.83E-07	up	0.33	1.08E-03	down
ENSMUSG00000118094	Gm52988	39.85	5.90E-03	up	0.05	1.89E-02	down
ENSMUSG00000118928	Gm25547	29.81	3.61E-02	up	0.04	3.84E-02	down
ENSMUSG00000119562	n-R5s130	136.33	9.78E-08	up	0.23	7.65E-03	down
ENSMUSG00000119648	Snord3b3	39.77	2.65E-02	up	0.03	2.65E-02	down
ENSMUSG00000119761	Snord3b2	41.75	2.28E-02	up	0.36	1.83E-02	down
ENSMUSG00000119851	Snord3b4	41.75	2.28E-02	up	0.08	2.02E-02	down
ENSMUSG00000119954	Gm35546	8.44	2.31E-05	up	0.03	2.39E-02	down

Table S10 Table of enrichment information for the 39°C HS-39°C DF group KEGG pathway

Number	Pathway id	Description	Ratio_in_study	Padjust	First Category	Second Category	Gene_names
1	mmu04659	Th17 cell differentiation	3/ 80	0.0163	Organismal Systems	Immune system	Fos;Rorc;Il2rb
2	mmu00350	Tyrosine metabolism	2/ 80	0.0167	Metabolism	Amino acid metabolism	Adh1;Aldh3a1
3	mmu05162	Measles	3/ 80	0.0375	Human Diseases	Infectious disease: viral	Hspa1b;Fos;Il2rb
4	mmu05134	Legionellosis	2/ 80	0.0384	Human Diseases	Infectious disease: bacterial	Hspa1b;Cxcl1
5	mmu00010	Glycolysis / Gluconeogenesis	2/ 80	0.0431	Metabolism	Carbohydrate metabolism	Adh1;Aldh3a1
6	mmu00750	Vitamin B6 metabolism	1/ 80	0.0448	Metabolism	Metabolism of cofactors and vitamins	Pdxp
7	mmu05020	Prion disease	4/ 80	0.0458	Human Diseases	Neurodegenerative disease	Hspa1b;Tuba8;mt-Atp8;Cacna1s
8	mmu00982	Drug metabolism - cytochrome P450	2/ 80	0.0479	Metabolism	Xenobiotics biodegradation and metabolism	Adh1;Aldh3a1
9	mmu00130	Ubiquinone and other terpenoid-quinone biosynthesis	1/ 80	0.0496	Metabolism	Metabolism of cofactors and vitamins	Nqo1
10	mmu00980	Metabolism of xenobiotics by cytochrome P450	2/ 80	0.0505	Metabolism	Xenobiotics biodegradation and metabolism	Adh1;Aldh3a1

Note: Pathway ID: Path number; Description: Specific description of KEGG pathway; Ratio in_study: The proportion of KEGG annotated genes in the target gene set that fall into the KEGG path. The numerator is the number of genes annotated into the KEGG path in the target gene set, and the denominator is the total number of genes annotated with KEGG in the target gene set; Pvalue: The uncorrected P-value, which represents whether the enriched result has statistical significance. The smaller the P-value, the more significant it is in statistics. Generally, a P-value less than 0.05 indicates a significant enrichment term for this function; First category: The branch name of the KEGG metabolic pathway.

Table S11 Table of enrichment information for the 41°C HS-41°C DF group KEGG pathway

Number	Pathway id	Description	Ratio_in_study	Pvalue	First Category	Second Category	Gene_names
1	mmu05020	Prion disease	5/37	0.0004	Human Diseases	Neurodegenerative disease	Hspa1b;Cox8a;Ndufa4l2;Hspa1l;Grin1
2	mmu04010	MAPK signaling pathway	4/37	0.0047	Environmental Information Processing	Signal transduction	Hspa1b;Cacnb4;Jund;Hspa1l
3	mmu00240	Pyrimidine metabolism	2/37	0.0076	Metabolism	Nucleotide metabolism	Tymp;Entpd3
4	mmu05134	Legionellosis	2/37	0.0090	Human Diseases	Infectious disease: bacterial	Hspa1b;Hspa1l
5	mmu04213	Longevity regulating pathway - multiple species	2/37	0.0090	Organismal Systems	Aging	Hspa1b;Hspa1l
6	mmu04260	Cardiac muscle contraction	2/37	0.0181	Organismal Systems	Circulatory system	Cox8a;Cacnb4
7	mmu05145	Toxoplasmosis	2/37	0.0274	Human Diseases	Infectious disease: parasitic	Hspa1b;Hspa1l
8	mmu05016	Huntington disease	3/37	0.0330	Human Diseases	Neurodegenerative disease	Grin1;Cox8a;Ndufa4l2
9	mmu04612	Antigen processing and presentation	2/37	0.0337	Organismal Systems	Immune system	Hspa1b;Hspa1l
10	mmu04915	Estrogen signaling pathway	2/37	0.0388	Organismal Systems	Endocrine system	Hspa1b;Hspa1l

Note: Pathway ID: Path number; Description: Specific description of KEGG pathway; Ratio in_study: The proportion of KEGG annotated genes in the target gene set that fall into the KEGG path. The numerator is the number of genes annotated into the KEGG path in the target gene set, and the denominator is the total number of genes annotated with KEGG in the target gene set; Pvalue: The uncorrected P-value, which represents whether the enriched result has statistical significance. The smaller the P-value, the more significant it is in statistics. Generally, a P-value less than 0.05 indicates a significant enrichment term for this function; First category: The branch name of the KEGG metabolic pathway.

Table S12 Table of enrichment information for the 43°C HS-43°C DF group KEGG pathway

Pathway id	Description	Ratio_in_study	Pvalue	First Category	Second Category	Gene_names
mmu05321	Inflammatory bowel disease	7/167	0.0000	Human Diseases	Immune disease	H2-DMa;Il13;H2-DMb2;Tgfb2;Il17f;Rora;Il12rb2
mmu05323	Rheumatoid arthritis	6/167	0.0003	Human Diseases	Immune disease	H2-DMa;Mmp3;Tgfb2;Flt1;H2-DMb2;Tnfsf13
mmu04640	Hematopoietic cell lineage	6/167	0.0005	Organismal Systems	Immune system	H2-DMa;Itgam;H2-DMb2;Gp5;Dntt;Itga2b
mmu04672	Intestinal immune network for IgA production	4/167	0.0011	Organismal Systems	Immune system	Tnfsf13;H2-DMb2;Pigr;H2-DMa
mmu04658	Th1 and Th2 cell differentiation	5/167	0.0024	Organismal Systems	Immune system	Il13;H2-DMb2;Dll1;H2-DMa;Il12rb2
mmu05150	Staphylococcus aureus infection	6/167	0.0016	Human Diseases	Infectious disease: bacterial	Fpr2;H2-DMa;Fpr1;Itgam;H2-DMb2;Krt12
mmu05310	Asthma	3/167	0.0023	Human Diseases	Immune disease	Il13;H2-DMb2;H2-DMa
mmu04657	IL-17 signaling pathway	5/167	0.0031	Organismal Systems	Immune system	Il13;Mmp3;Mmp9;Il17f;Mmp13
mmu04512	ECM-receptor interaction	5/167	0.0022	Environmental Information Processing	Signaling molecules and interaction	Itga2b;Tnn;Vtn;Gp5;Col4a4
mmu04510	Focal adhesion	7/167	0.0052	Cellular Processes	Cellular community - eukaryotes	Vtn;Tnn;Flt1;Col4a4;Itga2b;Mylk4;Kdr

Note: Pathway ID: Path number; Description: Specific description of KEGG pathway; Ratio in_study: The proportion of KEGG annotated genes in the target gene set that fall into the KEGG path. The numerator is the number of genes annotated into the KEGG path in the target gene set, and the denominator is the total number of genes annotated with KEGG in the target gene set; Pvalue: The uncorrected P-value, which represents whether the enriched result has statistical significance. The smaller the P-value, the more significant it is in statistics. Generally, a P-value less than 0.05 indicates a significant enrichment term for this function; First category: The branch name of the KEGG metabolic pathway.

Table S13 Enrichment statistics of gene set and metabolic set KEGG pathway regulated by heat stress in mung bean polyphenols 39°C MODE-K cells

Pathway ID	Pathway Description	First Category	Second Category	Metabolite number	Gene number
mmu05030	Cocaine addiction	Human Diseases	Substance dependence	L-Glutamate ; L-Tyrosine	-
mmu04068	FoxO signaling pathway	Environmental Information Processing	Signal transduction	L-Glutamate ; ADP	-
mmu00563	Glycosylphosphatidylinositol (GPI)-anchor biosynthesis	Metabolism	Glycan biosynthesis and metabolism	PE(18:3(6Z,9Z,12Z)/P-18:1(9Z)) ; PE(18:1(11Z)/18:1(11Z)) ; PE(18:1(11Z)/18:0) ; Uridine diphosphate-N-acetylglucosamine	-
mmu04659	Th17 cell differentiation	Organismal Systems	Immune system	-	Fos; Rorc ; Il2rb
mmu00380	Tryptophan metabolism	Metabolism	Amino acid metabolism	-	Adh1; Aldh3a1
mmu05162	Measles	Human Diseases	Infectious disease: viral	-	Hspa1b ; Fos; Il2rb
mmu05134	Legionellosis	Human Diseases	Infectious disease: bacterial	-	Hspa1b ;Cxcl1
mmu00010	Glycolysis / Gluconeogenesis	Metabolism	Carbohydrate metabolism	-	Adh1; Aldh3a1
mmu00750	Vitamin B6 metabolism	Metabolism	Metabolism of cofactors and vitamins	-	Pdxp
mmu05020	Prion diseases	Human Diseases	Neurodegenerative disease	-	Hspa1b ; Tuba8; mt-Atp8 ; Cacna1s
mmu00983	Drug metabolism - other enzymes	Metabolism	Xenobiotics biodegradation and metabolism	-	-

mmu00130	Ubiquinone and other terpenoid-quinone biosynthesis	Metabolism	Metabolism of cofactors and vitamins	-	Nqo1
mmu05140	Leishmaniasis	Human Diseases	Infectious disease: parasitic	PS(18:1(9Z)/18:0) ; Cer(d18:1/24:1(15Z))	-
mmu05231	Choline metabolism in cancer	Human Diseases	Cancer: overview	GPCho(18:1/16:0) ; LysoPC(20:4(8Z,11Z,14Z,17Z)/0:0) ; PC(18:0/18:3(9Z,12Z,15Z)) ; PC(14:0/14:0) ; PC(18:1(11Z)/14:1(9Z)) ; PC(16:1(9Z)/14:0) ; PC(18:0/0:0) ; PC(16:0/18:2(9Z,12Z))	-

Table A14 Mung bean polyphenols 41°C MODE-K cells heat stress regulated metabolic set and gene set KEGG pathway enrichment

Pathway ID	Pathway Description	First Category	Second Category	Gene number	Gene list
mmu00230	Purine metabolism	Metabolism	Nucleotide metabolism	2	Entpd3 ; Pde6a
mmu05016	Huntington disease	Human Diseases	Neurodegenerative disease	3	Grin1 ; Cox8a ; Ndufa4l2
mmu05145	Toxoplasmosis	Human Diseases	Infectious disease: parasitic	2	Hspa1b; Hspa1l
mmu04915	Estrogen signaling pathway	Organismal Systems	Endocrine system	2	Hspa1b ; Hspa1l
mmu03040	Spliceosome	Genetic Information Processing	Transcription	2	Hspa1b ; Hspa1l
mmu04612	Antigen processing and presentation	Organismal Systems	Immune system	2	Hspa1b ; Hspa1l
mmu05162	Measles	Human Diseases	Infectious disease: viral	2	Hspa1b ; Hspa1l
mmu00190	Oxidative phosphorylation	Metabolism	Energy metabolism	2	Cox8a ; Ndufa4l2
mmu04260	Cardiac muscle contraction	Organismal Systems	Circulatory system	2	Cox8a ; Cacnb4
mmu00240	Pyrimidine metabolism	Metabolism	Nucleotide metabolism	2	Tymp ; Entpd3

mmu04010	MAPK signaling pathway	Environmental Information Processing	Signal transduction	4	Hspa1b; Cacnb4 ; Jund; Hspa1l
mmu04213	Longevity regulating pathway - multiple species	Organismal Systems	Aging	2	Hspa1b ; Hspa1l
mmu05134	Legionellosis	Human Diseases	Infectious disease: bacterial	2	Hspa1b ; Hspa1l
mmu05020	Prion diseases	Human Diseases	Neurodegenerative disease	5	Hspa1b ; Cox8a ; Ndufa4l2; Hspa1l;Grin1

Table A15 Enrichment statistics of gene set and metabolic set KEGG pathway regulated by heat stress in mung bean polyphenols 43°C MODE-K cells

Pathway ID	Pathway Description	First Category	Second Category	Metabolite number	Gene list
mmu04080	Neuroactive ligand-receptor interaction	Environmental Information Processing	Signaling molecules and interaction	-	Hrh4; Fpr2; Fpr1; Gria4; Gpr83; Cckar; Avp ; Grin3a
mmu04060	Cytokine-cytokine receptor interaction	Environmental Information Processing	Signaling molecules and interaction	-	Amh; Il13; Eda2r; Tgfb2; Il17f; Tnfsf13; Il12rb2
mmu04151	PI3K-Akt signaling pathway	Environmental Information Processing	Signal transduction	-	Vtn; Tnn; Fgfr3; Flt1; Col4a4; Itga2b; Areg; Kdr
mmu04659	Th17 cell differentiation	Organismal Systems	Immune system	-	H2-DMb2; H2-DMa;Rora; Il17f
mmu04015	Rap1 signaling pathway	Environmental Information Processing	Signal transduction	-	Fpr1; Itgam; Fgfr3; Flt1; Itga2b; Kdr
mmu04928	Parathyroid hormone synthesis, secretion and action	Organismal Systems	Endocrine system	-	Slc34a3; Mmp13; Bglap; Mmp25
mmu05418	Fluid shear stress and atherosclerosis	Human Diseases	Cardiovascular disease	-	Itga2b; Kdr; Mmp9; Sele; Gstm6
mmu04933	AGE-RAGE signaling pathway in diabetic complications	Human Diseases	Endocrine and metabolic disease	-	Plcd4; Tgfb2; Sele; Col4a4
mmu05202	Transcriptional misregulation in cancer	Human Diseases	Cancer: overview	-	Itgam; Mmp3; Mmp9; Flt1; Mpo; Tspan7

mmu00380	Tryptophan metabolism	Metabolism	Amino acid metabolism	-	Ehhadh; Tph1; Inmt
mmu04020	Calcium signaling pathway	Environmental Information Processing	Signal transduction	-	Fgfr3; Flt1; Cckar ; Plcd4; Mcoln3; Mylk4; Kdr
mmu05140	Leishmaniasis	Human Diseases	Infectious disease: parasitic	-	Itgam; H2-DMb2; H2-DMa; Tgfb2
mmu04510	Focal adhesion	Cellular Processes	Cellular community - eukaryotes	-	Vtn; Tnn; Flt1; Col4a4; Itga2b; Mylk4; Kdr
mmu04512	ECM-receptor interaction	Environmental Information Processing	Signaling molecules and interaction	-	Itga2b; Tnn; Vtn; Gp5; Col4a4
mmu04657	IL-17 signaling pathway	Organismal Systems	Immune system	-	Il13; Mmp3; Mmp9; Il17f; Mmp13
mmu05310	Asthma	Human Diseases	Immune disease	-	Il13; H2-DMb2; H2-Dma
mmu05150	Staphylococcus aureus infection	Human Diseases	Infectious disease: bacterial	-	Fpr2; H2-DMa; Fpr1; Itgam; H2-DMb2; Krt12
mmu04658	Th1 and Th2 cell differentiation	Organismal Systems	Immune system	-	Il13; H2-DMb2; Dll1; H2-DMa; Il12rb2
mmu04672	Intestinal immune network for IgA production	Organismal Systems	Immune system	-	Tnfsf13; H2-DMb2; Pigr; H2-Dma
mmu04640	Hematopoietic cell lineage	Organismal Systems	Immune system	-	H2-DMa; Itgam; H2-DMb2; Gp5; Dntt; Itga2b
mmu05323	Rheumatoid arthritis	Human Diseases	Immune disease	-	H2-DMa; Mmp3; Tgfb2; Flt1; H2-DMb2; Tnfsf13
mmu05321	Inflammatory bowel disease (IBD)	Human Diseases	Immune disease	-	H2-DMa; Il13;H2-DMb2; Tgfb2; Il17f; Rora; Il12rb2
mmu04723	Retrograde endocannabinoid signaling	Organismal Systems	Nervous system	PGH2 ; PE(16:1(9Z)/15:0) ; PC(14:0/14:0)	-
mmu05231	Choline metabolism in cancer	Human Diseases	Cancer: overview	PC(14:0/14:0) ; Citicoline	-
